# SKP2 promotes breast cancer tumorigenesis and radiation tolerance through PDCD4 ubiquitination

**DOI:** 10.1186/s13046-019-1069-3

**Published:** 2019-02-13

**Authors:** Ce Li, Lutao Du, Yidan Ren, Xiaoyan Liu, Qinlian Jiao, Donghai Cui, Mingxin Wen, Chuanxin Wang, Guangwei Wei, Yunshan Wang, Aiguo Ji, Qin Wang

**Affiliations:** 1 School of Pharmaceutical Sciences, Shandong University, 44 Wenhua Xi Road, Jinan, 250012 Shandong China; 2 Department of Clinical Laboratory, The Second Hospital of Shandong University, 247 Beiyuan Street, Tianqiao District, Jinan, 250033 Shandong China; 3 International Biotechnology R&D Center, Shandong University School of Ocean, 180 Wenhua Xi Road, Weihai, 264209 Shandong China; 4 Department of Human Anatomy and Key Laboratory of Experimental Teratology, Ministry of Education, Shandong University School of Medicine, 44 Wenhua Xi Road, Jinan, 250012 Shandong China; 5 Department of Anesthesiology, Qilu Hospital, Shandong University, 107 Wenhua Xi Road, Jinan, 250012 China

**Keywords:** Breast cancer, SKP2, PDCD4, Cell apoptosis, DNA-damage response, Radiotherapy

## Abstract

**Background:**

S-phase kinase-associated protein 2 (SKP2) is an oncogene and cell cycle regulator that specifically recognizes phosphorylated cell cycle regulator proteins and mediates their ubiquitination. Programmed cell death protein 4 (PDCD4) is a tumor suppressor gene that plays a role in cell apoptosis and DNA-damage response via interacting with eukaryotic initiation factor-4A (eIF4A) and P53. Previous research showed SKP2 may interact with PDCD4, however the relationship between SKP2 and PDCD4 is unclear.

**Methods:**

To validate the interaction between SKP2 and PDCD4, mass spectrometric analysis and reciprocal co-immunoprecipitation (Co-IP) experiments were performed. SKP2 stably overexpressed or knockdown breast cancer cell lines were established and western blot was used to detect proteins changes before and after radiation. In vitro and in vivo experiments were performed to verify whether SKP2 inhibits cell apoptosis and promotes DNA-damage response via PDCD4 suppression. SMIP004 was used to test the effect of radiotherapy combined with SKP2 inhibitor.

**Results:**

We found that SKP2 remarkably promoted PDCD4 phosphorylation, ubiquitination and degradation. SKP2 promoted cell proliferation, inhibited cell apoptosis and enhanced the response to DNA-damage via PDCD4 suppression in breast cancer. SKP2 and PDCD4 showed negative correlation in human breast cancer tissues. Radiotherapy combine with SKP2 inhibitor SMIP004 showed significant inhibitory effects on breast cancer cells in vitro and in vivo.

**Conclusions:**

We identify PDCD4 as an important ubiquitination substrate of SKP2. SKP2 promotes breast cancer tumorigenesis and radiation tolerance via PDCD4 degradation. Radiotherapy combine with SKP2-targeted adjuvant therapy may improve breast cancer patient survival in clinical medicine.

**Electronic supplementary material:**

The online version of this article (10.1186/s13046-019-1069-3) contains supplementary material, which is available to authorized users.

## Backgrounds

Breast cancer ranks first in female cancers, which resulted in 5.22 million deaths at 2013 [1]. Radiotherapy is an important component for early breast cancer treatment [2]. Postoperative radiotherapy can reduce local tumor recurrence rates, as well as improve the long-term survival of patients and local tumor control [3]. S-phase kinase-associated protein 2 (SKP2), also known as p45 or FBXL1, is one of the members of the F-box protein family, which participates in ubiquitination, cell cycle control and signal transduction in the form of the SKP2-SCF complex (Cul1-Rbx1-SKP1-F-box^SKP2^) [4, 5]. In the SCF complex, SKP2 plays a role as a substrate recognition factor [6]. SKP2 has been proved as an oncogene [7], which is overexpressed in lymphoma [8], prostate cancer [9], melanoma [10], nasopharyngeal carcinoma [11], breast cancer [12] and so on. Recent studies have shown that SKP2 also plays an important role in DNA-damage repair. SKP2 E3 ligase is involved in MRN complex-mediated ATM activation by triggering K63-linked ubiquitination of NBS1 in response to DNA double-strand breaks (DSBs). Increased radiation sensitivity appears in SKP2-deficient cells, which exhibit a defect in homologous recombination (HR) repair [13]. However, a detailed understanding of the mechanisms of SKP2 in DNA-damage response is still lacking.

Programmed cell death protein 4 (PDCD4), which is known as a tumor suppressor gene, is down-expressed or deficient in a range of tumors, including breast cancer [14], colorectal cancer [15], glioma [16] and hepatocellular carcinoma [17]. PDCD4 suppresses tumors by inhibiting protein initiation complex formation. PDCD4 can combine with eukaryotic initiation factor-4A (eIF4A), thereby inhibiting its enzymatic activity, leaving the mRNA methylated decapping process unfinished and eIF4A-eIF4G complex unformed, thus inhibiting proliferation of tumor cells [18, 19]. PDCD4 participates in DNA-damage response by inhibiting the P53 protein translation process [20]. PDCD4 knockout cells show an increased sensitivity and survival to agents that cause DNA-damage, such as UV light, etoposide or ethyl-methanesulfonate [21]. PDCD4 is rapidly phosphorylated on Ser67 by the protein kinase S6K1 and subsequently degraded by ubiquitin ligase SCF^βTRCP^ in response to mitogens [22]. Expression of PDCD4(S67/71A) also induces a slower accumulation of SKP2 [22]. These results imply PDCD4 may be a degradation substrate of SCF^SKP2^.

In this study, we showed PDCD4 was a novel ubiquitination substrate of SCF^SKP2^. SCF^SKP2^ dramatically promoted PDCD4 phosphorylation via the AKT pathway, then triggered PDCD4 ubiquitination and degradation. Notably, we found SKP2 regulated apoptosis and DNA-damage response via PDCD4 suppression. PDCD4 also regulated SKP2 expression in reverse. Together, our findings reveal a new path of SKP2 promoting tumorigenesis and radiation tolerance through PDCD4 degradation, and also provide a possible mechanistic explanation for SKP2 acting as an oncogene. SMIP004, a SKP2 inhibitor, showed remarkable inhibitory effects after radiation in breast cancer by inducing cell apoptosis and inhibiting DNA-damage response. SMIP004 combined with radiotherapy showed better antitumor effects compared with radiotherapy alone. SKP2 inhibitors may be potential sensitizers for radiation therapy with important clinical value.

## Methods

### Mice, cell lines and cell culture

WT and SKP2^−/−^ MEF mice were purchased from Model Animal Research Center Of Nanjing University. 293 T, MDA-MB-231, MCF-7, MDA-MB-468, SKBR-3 cells were obtained from American Type Culture Collection and cultured in DMEM medium containing 10% foetal bovine serum (FBS).

### Expression plasmids

lentiCRISPR v2, pCMV-VSV-G, psPAX, pcDNA3-myc-Skp2, CMV10-3xFlag Skp2 delta-F, pSuper-retro-puro, HA-Ubiquitin, pRK5-HA-Ubiquitin-K48R and pET3a-Ubiquitin-K63R plasmids were purchased from Addgene, Flag–His-SKP2, Flag-His-ΔN-SKP2, Flag-His-PDCD4 and Flag-His-PDCD4(Ser67)plasmids were purchased from Biosune Biotechnology. The sgRNA and shRNA sequences for SKP2 were as follows: sgSKP2:AGAATCCAGAACACCCAGAA; shSKP2:GATCCCCGCCTAAGCTAAATCGAGAGAATTCAAGAGATTCTCTCGATTTAGCTTAGGCTTTTTGGAAA. shRNA sequences for PDCD4 were as follows: CCGGGCGGTTTGTAGAAGAATGTTTCTCGAGAAACATTCTTCTACAAACCGCTTTTTG.

### Radiation of cells and nude mice

Cells were transferred to 6-cm^2^ flasks and incubated in DMEM with 10% FBS at 37 °C with 5% CO_2_ for 24 h. The flasks were placed on a linear accelerator PRIMUS H (SIEMENS, GER) with a fixed source skin distance and X-ray irradiation at 0.6GY/min for 10 min. Nude mice were exposed to whole body-irradiationa at 0.1GY/min for 10 min twice a week at 4–6 weeks after tumor cells injection.

### Antibodies and reagents

The following antibodies were used for IP or immunoblotting (IB): SKP2 antibody (IP:1:200, IB: 1:1000, Cell Signaling Technology, #2652), PDCD4 antibody (IP: 1:200, IB: 1:1000, Cell Signaling Technology, #9535), phosphorylated PDCD4(Ser67) antibody (IP: 1:200, IB: 1:1000, Abcam, ab73343), Flag antibody (IP: 1:200; IB:1:1000, Cell Signaling Technology, F1804), Caspase3 antibody (IB: 1:1000, Cell Signaling Technology, #9662), Cleaved Caspase3 antibody (IB: 1:1000, Cell Signaling Technology, #9664), γ-H2AX antibody (IB: 1:1000, Abcam, ab26350), HA-Tag antibody (IB,1:1000; Cell Signaling Technology, #3724), Myc-tag antibody (IP,1:200, IB,1:1000; Cell Signaling Technology, #2276), AKT(AKT3 + AKT2 + AKT1) antibody (IB:1:5000, Abcam, ab32505), pAKT(AKT3 (phospho S472) + AKT2 (phospho S474) + AKT1 (phospho S473)) antibody (IB:1:5000, Abcam, ab192623), PCNA antibody (IB,1:2000; Cell Signaling Technology, #2276), P53 antibody (IB: 1:1000, Abcam, ab32389), phosphorylated P53(Ser15) antibody (IB: 1:1000, Abcam, ab1431), Bax antibody (IB,1:1000; Cell Signaling Technology, #2772), Bcl-2 antibody (IB,1:1000; Cell Signaling Technology, #2872), β-Actin antibody (IB: 1:1000, Cell Signaling Technology, #3700), GAPDH antibody (IB: 1:1000, Abcam, ab32389), CHX, MG132 and RIPA Lysis Buffer were from Beyotime Biotechnology. SKP2 inhibitor SMIP004 and AKT inhibitor MK-2206 were from MCE. Protease inhibitor cocktai was from Roche. lipo-2000 were from Invitrogen.

### Quantitative real-time PCR

RNA was isolated using RNeasy reagents (Thermo, USA) and then transcribed into cDNA using SuperscriptIII reagents (Invitrogen) with an oligo(dT)_20_ primer. Quantitative real-timePCR was performed using Power SYBR Green Mastermix (Thermo, USA) on an Eppendorf realplex^2^ Real-Time PCR System. All oligonucleotide primers were obtained from GENEWIZ Technologies (Suzhou, China). The housekeeping gene GAPDH was used as loading controls. The sequences of primer sets were 5′- TTGCCCTGCAGACTTTGCTA -3′ and 5’-CAGCTGGGTGATGGTCTCTG-3′ for SKP2; 5′- TGGATTAACTGTGCCAACCA-3′ and 5′- TCTCAAATGCCCTTTCATCC-3′ for PDCD4; 5’-GTCTCCTCTGACTTCAACAGCG-3′ and 5’-ACCACCCTGTT GCTGTAGCCAA-3′ for GAPDH.

### Establish SKP2 stably overexpressing MCF-7 breast cancer cell line

The number of MCF-7 cells was 1 × 10^6^ /well in 6-well plates before transfection, 14 μg of pcDNA3-myc-SKP2 plasmid and 10 μL of Lipofectamine 2000 were transfected into MCF-7 cells according to the instructions of Lipofectamine 2000 Transfection Reagent. The cells were screened by using G418, and resistant clones were visualized after about 2 weeks of screening, followed by further monoclonalization of the cells. The result was verified by western blot.

### Establish SKP2 stably silencing MDA-MB-231 breast cancer cell line

The pSuper-retro-puro plasmid was linearized with BglII and HindIII enzyme (NEB, USA) and linearized with the verified shSKP2 sequence(from Sigma website) to construct the pSuper-retro-puro-shSKP2 recombinant plasmid and diluted to about 1 μg/μl; then transfected into 293 T cells with calcium phosphate for 48 h and the retrovirus was producted in supernatant. MDA-MB-231 cells was continuously by retrovirus infected for 3 days, and the positive cells were screened for 2 weeks with the medium containing puromycin (0.5 μg/ml). The result was verified by western blot.

### Establish stably SKP2 Cas-9 knockout MDA-MB-231 breast cancer cell line

The SKP2 Cas-9 gene knockout plasmid was constructed using the Lenti CRISPR V2 plasmid, then transfected with psPAX2 and pCMV-VSV-G plasmid at ratio of 4:3:2 into 293 T cells. MDA-MB-231 cells in 60 mm dish were infected with 1 to 5 mL of virus supernatant when the cell density is 60–80%. 50 to 100 cells were evenly divided into 96-well plates and cultured, and the cells were directly subjected to PCR, then sequenced, and the successfully silenced cells were expanded and cultured. The result was verified by western blot.

### Establish PDCD4 stably overexpressing MCF-7-SKP2 breast cancer cell line and PDCD4 stably silencing MDA-MB-231-sgSKP2 breast cancer cell line

The method is the same as above. 14 μg pcDNA3-Flag-PDCD4 plasmid and 10 μL of Lipofectamine 2000 were transfected into MCF-7-SKP2 cells, then screened by G418 for 14 days. pSuper-retro-puro-shPDCD4 recombinant plasmid was diluted to about 1 μg/μl and then transfected into 293 T cells with calcium phosphate for 48 h and the retrovirus was producted in supernatant. MDA-MB-231-sgSKP2 cells were continuously infected by retrovirus for 3 days, and the positive cells were screened with puromycin for 2 weeks. The result was verified by western blot.

### Immunoblots and immunoprecipitation

For western bloting, Whole-cell lysates were prepared using RIPA lysis buffer supplemented with protease inhibitors. For immunoprecipitation, Whole-cell lysates were prepared using weak RIPA lysis buffer supplemented with protease inhibitors. 1000 μg protein and 5 μL antibody were incubated in 200 μL immunoprecipitation buffer for 4 h before 20 μL immunomagnetic beads (Millipore) were added overnight, followed by western bloting which was performed as described previously [23].

### Mass spectrometry and protein identification

Two hundred ninety-three T cells stably transfected with vector or Myc-SKP2 were immunoprecipitated with Myc antibody, and subjected to SDS–PAGE. Protein bands were excised from Commassie blue staining. Proteins were identified and searched against the NCBI protein database.

### Ubiquitination assay in vitro and in vivo

In vivo ubiquitination assays were performed as described [24]. In brief, 293 T cells were transfected with the indicated plasmids for 48 h and subjected to IB analysis. For in vitro ubiquitination assays, Flag–SKP2 and PDCD4 proteins purified from the 293 T cells were eluted in SDS-sample buffer and immunoblotted with anti-PDCD4 antibody.

### GST pull-down assays

For in vitro GST–SKP2 and PDCD4/PDCD4 (Ser67) interaction, GST–SKP2 proteins were purified from the bacterial lysates of BL21 competent cells using the glutathione-agarose beads according to the manufacturer’s standard procedures.Then the GST–SKP2 proteins bound to glutathione beads were incubated with the in vitro translated PDCD4 or PDCD4 (Ser67) peptide fragments at 4 °C overnight in the interaction buffer four times, and subjected to 8% SDS–PAGE, followed by IB.

### MTT assay

MTT assay was performed to assess cell proliferation. When the cell density was about 70%, digested and adjusted the cell density at 1.5 × 10^4^ cells/mL, inoculated 100 μL/well into 96-well plates, set up 3 replicate wells for each cell line; After 12, 24, 48, 72 and 96 h, added 5 μL of 5 mg/mL 3-[4, 5-dimethylthiazol-2-yl]-2, 5 diphenyltetrazolium bromide (MTT) per well, continued to culture for 4 h; carefully aspirated the supernatant, added 200 μL DMSO per well, shaked for 10 min in a horizontal shaker, and measured OD value at 490 nm.

### Colony formation assay

Colony formation assays were performed to assess cell proliferation. Cells in logarithmic growth phasewas digested and inoculated 800 cells in each 60 mm culture dish, set up 3 replicate dishes for each cell line cultured for 2 weeks in cell incubator; aspirated the supernatant, washed 3 times with PBS; Add 10 mL of methanol to the dish and fixed for 30 min; removed the methanol, added 1:10 dilution of Giemsa dye solution and kept for 30 min, rinsed off the dye solution with water, and dried naturally; counted, the number of cells above 30 was recorded as a clone, the number of clones was counted under the microscope and statistical analysis was performed.

### Cell apoptosis assay

Annexin V-APC/7-AAD apoptosis kit Flow was purchased from BD Bioscience (USA, Catalogue No. 550474). Washed cells twice with cold PBS and then resuspended cells in 1X Binding Buffer at a concentration of 1 × 10^6^ cells/ml. Transfered 100 μl of the solution (1 × 10^5^ cells) to a 5 ml culture tube. Added 5 μl of APC Annexin V (for one and two color analysis) and 5 μl of 7-AAD (for two color analysis only). Gently vortexed the cells and incubated for 15 min at RT (25 °C) in the dark. Added 400 μl of 1X Binding Buffer to each tube. Analyzed by flow cytometry within 1 h.

### Hoechst 33342 staining

Hoechst 33342/PI (propidine iodide) apoptosis and Necrosis Assay Kit was purchased from Beyotime (China, Catalogue No. C1056). Washed cells twice with cold PBS and added 5 μl of Hoechst 33342 staining solution. Mixed gently, incubated at 4 °C for 20–30 min in an ice bath. Then observed with a microscope ant took photos.

### In vivo tumorigenesis assay

For in vivo tumorigenesis assays, MCF-7, MCF-7-Con, MCF-7-SKP2, MCF7-SKP2-PDCD4 and MDA-MB-231, MDA-MB-231-Con, MDA-MB-231-sgSKP2, MDA-MB-231-sgSKP2-shPDCD4 stable expressed cells were cultured and ogarithmic growth phase cells were digested and resuspended in PBS, adjusted cell density to 5 × 10^6^/ml, subcutaneous injection 0.1 ml into the left lank of 6-week-old nude mice. Tumor size was measured weekly using acaliper, and the tumor volume was determined with the standard formula: L× W^2^ × 0.5^2^, where L is the longest diameter and W is the shortest diameter. After about 6 weeks, tumors were taken, the tumor weight and volume were determined. The Institutional Animal Care and Use Committee (IACUC) is from Ethics Committee of Qilu Hospital of Shandong University.

### Patients and human materials

The institutional review board of QiLu hospital had approved the study by using formalin-fixed tissue. IHC analysis was performed on cancer and corresponding non-cancerous tissues from 60 breast carcinoma patients and 40 liver cancer patients between 2012 and 2015.

### IHC and scoring

The procedures of IHC studies were performed as previously described [23]. In brief, The samples were fixed with 10% formalin, embedded in paraffin and sliced into 5-μm sections. The slides were incubated with primary antibodies against SKP2 (Cell Signaling Technology, 1:100), PDCD4 (Cell Signaling Technology, 1:50), Caspase-3 (Cell Signaling Technology, 1:100), γ-H2AX (Abcam, 1:100) and Ki-67 antibody (Epitomics, 1:160). Staining was observed in 5 randomly selected high-power fields. The staining intensity was based on the average percentage of positive cells. The scoring results were analyzed by 2 investigators.

### Statistical analysis

Results were expressed as mean ± SD from at least three independent experiments. SPSS19.0 statistical software package (SPSS Inc.) was used for statistical analysis. Statistical differences between groups were assessed using the Student *t* test. Association between SKP2 and PDCD4 expression in breast cancer cell lines was evaluated by the Spearman rank correlation test. Association between SKP2 and PDCD4 expression in colorectal cancer tissue was evaluated by the Chi-square test. *P* < 0.05 was considered statistically significant.

## Results

### SKP2 interacts with PDCD4 and negatively regulates PDCD4 protein levels

**Fig. 1:**
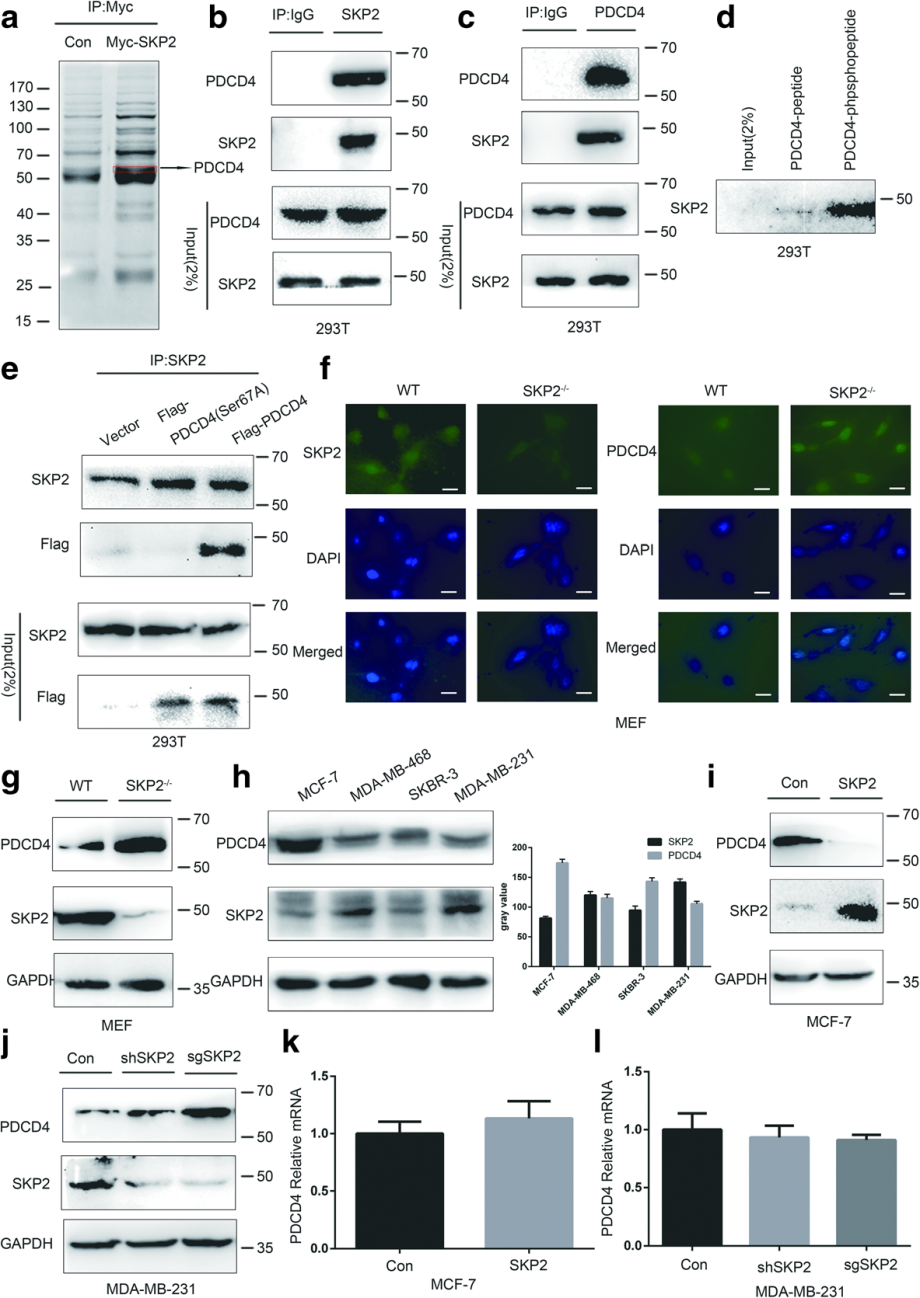
SKP2 binds to PDCD4 and negatively regulates PDCD4 protein levels. **a** Lysates from 293 T cells stably transfected with vector or Myc-SKP2 were immunoprecipitated with Myc antibody and subjected to mass spectrometry analysis. PDCD4 was identified as a novel binding partner for SKP2. **b**, **c** 293 T cells were harvested for immunoprecipitation with SKP2 antibody (b) or PDCD4 antibody (**c**), followed by immunoblotting. **d** 35S-labeled in vitro translated SKP2 was used in binding reactions with beads coupled to the PDCD4 peptide K^65^NSSRDSGRGDSVSD^79^ or the phosphopeptide K^65^NSpSRDSGRGDSVSD^79^. Bound proteins were eluted and subjected to electrophoresis and autoradiography. **e** 293 T cells were transfected with indicated plasmids and harvested for immunoprecipitation assay. **f** SKP2 and PDCD4 expression of SKP2^−/−^ and WT MEF cells were determined by immunostaining (Scale bars, 10 um). **g** PDCD4 protein levels were increased in SKP2^−/−^ MEFs compared with WT MEFs. **h** PDCD4 and SKP2 protein expression levels in breast cancer cell lines were determined by IB and gray values were analyzed. Spearman rank correlation test was used to test of correlation between the SKP2 and PDCD4 protein expression levels, r = − 1.00, *p* < 0.01. **i** Overexpression of SKP2 decreased the levels of PDCD4 protein in MCF-7 cells: MCF-7 cells were transfected with SKP2 or vector control, followed by IB for protein expression. **j** SKP2 silencing increased the endogenous levels of PDCD4 protein in MDA-MB-231 cells: MDA-MB-231 cells were transfected with sgRNA and shRNA targeting SKP2, followed by IB for protein expression. **k** MCF-7 cells were transfected with SKP2 or vector control, followed by qRT-PCR for mRNA expression. **l** MDA-MB-231 cells were transfected with sgSKP2 or vector control, followed by qRT-PCR for mRNA expression. **h, k, l** Data represent the mean ± SEM of three independent experiments

Previous studies have shown that PDCD4 can be ubiquitylated and degraded by β-TRCP, a member of the F-box protein family. SKP2, also a F-box family member, showed correlation with PDCD4 [22]. We assumed that SKP2 may bind PDCD4 and mediate PDCD4 ubiquitination and degradation. To verify our inference, we generated 293 T cells stably overexpressed with Myc-SKP2, and the total cell lysates from these cells were immunoprecipitated with Myc antibody to pull down SKP2 interacting proteins. Mass spectrometric analysis was used to indicate that several peptides existed in the SKP2 immunocomplex, one of which corresponded to PDCD4 (Fig. 1a).

To validate the interaction between SKP2 and PDCD4, reciprocal co-immunoprecipitation (Co-IP) experiments were performed. We found that endogenous SKP2 interacted with endogenous PDCD4 (Fig. 1b, c). Previous research has shown that SCF^SKP2^ specifically binds to the phosphorylated form of protein and targets it for degradation through a ubiquitin-dependent process [25]. Exogenous wild-type (WT) PDCD4 immunoprecipitated efficiently with SKP2, but PDCD4(S76A) mutant did not [22]. We then studied whether there was a similar phenomenon between SKP2 and PDCD4. Surprisingly, a peptide (amino acids 65–79) from PDCD4 containing phosphorylated Ser67 efficiently bounded to SKP2, but a corresponding non-phosphorylated peptide did not (Fig. 1d). PDCD4 (S67A) mutant also failed to bind to SKP2 in vivo (Fig. 1e). We further demonstrated that SKP2 interacted with PDCD4 in the form of SCF^SKP2^ complex by using Co-IP assays. F-box domain and C-terminal LRR domain (amino acids 201–424) were also required for the interaction (Additional file 1: Figure S1a).

Having detected a physical interaction between the two proteins, we next determined whether PDCD4 protein levels were regulated by SKP2. We found that PDCD4 protein levels were upregulated in primary SKP2^−/−^ MEF cells compared with WT MEF cells by using immunofluorescence and western blot (Fig. 1f, g). The results were also verified in SKP2^−/−^ and WT mouse tissues by IHC (Additional file 2: Figure S2a). SKP2 and PDCD4 showed opposite expression levels in human breast cell lines (Fig. 1h). SKP2 was highly expressed in MDA-MB-231 and MDA-MB-468 cell lines, whereas lowly expressed in MCF-7 and SKBR-3 cell lines. PDCD4 showed opposite expression in these cell lines. The levels of PDCD4 were reduced upon SKP2 transfection and overexpressed upon SKP2 knockdown (Fig. 1i, j). However, overexpression of SKP2ΔF had no effect (Additional file 1: Figure S1b). Furthermore, SKP2 had no effect on the levels of PDCD4 mRNA (Fig. 1k, l), suggesting SKP2 promoted PDCD4 protein degradation. Collectively, these data strongly suggest that PDCD4 could be a novel substrate of SKP2.

### SCF^SKP2^ promotes PDCD4 phosphorylation, ubiquitination and degradation

**Fig. 2:**
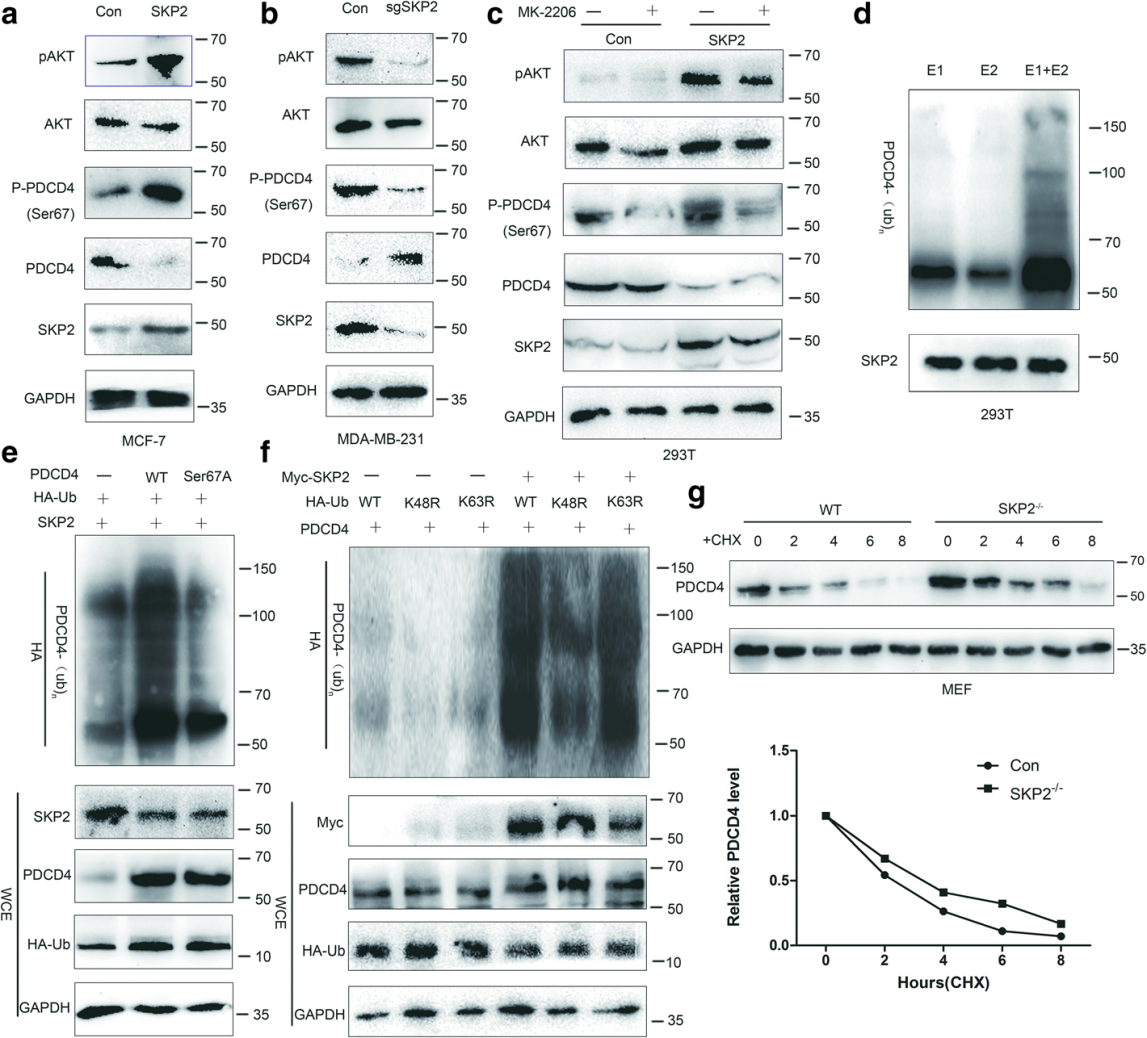
SCF^SKP2^ is a direct E3 ligase for PDCD4 and triggers phosphorylation, ubiquitination and degradation of PDCD4. **a** MCF-7 cells were transfected with Flag-SKP2 and control plasmid for 48 h and harvested for IB. **b** MDA-MB-231 cells were transfected with sgSKP2 and control plasmid for 48 h and harvested for IB. **c** 293 T cells were transfected with Flag-SKP2 and control plasmid for 48 h. Cells were switched to fresh medium (10% FBS) containing AKT inhibitor MK-2206 (2.5 μM) 24 h or not post transfection and harvested for IB. **d** Flag–SKP2 proteins isolated 293 T cells transfected with Flag–SKP2 were incubated with ATP, E1, E2 along with PDCD4 proteins isolated from 293 T cells for in vitro ubiquitination assay. **e** In vivo ubiquitination assay from 293 T cells transfected with HA-Ubiquitin (HA-Ub) and indicated plasmids. **f** In vivo ubiquitination assay of 293 T cells transfected with HA-Ubiquitin (HA-Ub), HA-Ub K48R or HA-Ub K63R and indicated plasmids. **g** PDCD4 half-life is extended in SKP2^−/−^ MEFs compared with SKP2 WT MEFs. Cells were switched to fresh medium (10% FBS) containing cycloheximide (CHX) 48 h post transfection for indicated time periods and harvested for IB. The band density was quantified

Previous research has shown that AKT causes PDCD4 phosphorylation at Ser67, which affects the protein stability of PDCD4 [26]. SKP2 overexpression correlates with AKT activation, and AKT mediates downstream substrates phosphorylation [26, 27]. SCF^SKP2^ directly triggers ubiquitination and degradation of its phosphorylated protein substrates [28, 29]. Since SKP2 regulated PDCD4 protein levels, it is highly possible that SKP2 plays a role in the phosphorylation, ubiquitylation and degradation of PDCD4. To test this hypothesis, we explored whether SKP2 could upregulate PDCD4 phosphorylation levels. Western blot results showed that phosphorylated PDCD4 (Ser67) and pAKT levels were remarkably increased upon SKP2 transfection (Fig. 2a). On the other hand, transfection of sgRNA targeting SKP2 led to a decrease in phosphorylated PDCD4 (Ser67) and pAKT levels (Fig. 2b). AKT inhibitor MK-2206 reversed the impact on phosphorylated PDCD4 levels in SKP2 transfected cells (Fig. 2c), indicating that SKP2 upregulates PDCD4 phosphorylation levels through the AKT signal pathway.

To verify the conjecture that SCF^SKP2^ is a direct E3 ligase for PDCD4, we performed in vitro ubiquitination assays and found that SCF^SKP2^ could induce PDCD4 ubiquitination in vitro (Fig. 2d). Then we conducted in vivo ubiquitination assays to determine whether SKP2 promotes PDCD4 ubiquitination. Notably, overexpression of SKP2 promoted ubiquitination of PDCD4 in the presence of the proteasome inhibitor MG132. PDCD4 (Ser67A) was unable to be ubiquitinated by SKP2, suggesting phosphorylation of PDCD4 on Ser67 is a requirement for PDCD4 ubiquitination (Fig. 2e). We further demonstrated that SKP2 triggered K48-linked ubiquitination of PDCD4 (Fig. 2f). Accordingly, these results suggest that PDCD4 is a novel substrate for SKP2. K48-linked ubiquitination is linked to proteasome-dependent degradation [30]. We then determined whether SKP2 could affect PDCD4 protein half-life. Indeed, SKP2^−/−^ extended PDCD4 protein half-life, leading to its stabilisation (Fig. 2g). These means SKP2 promotes K48-linked ubiquitination of PDCD4 in a proteasome-dependent manner. Therefore, our results suggest that SCF^SKP2^ is the E3 ligase for PDCD4 that promotes PDCD4 ubiquitination and degradation.

### PDCD4 negatively regulate SKP2 protein levels and SKP2 increases the survival of breast cancer cells via PDCD4 suppression

**Fig. 3:**
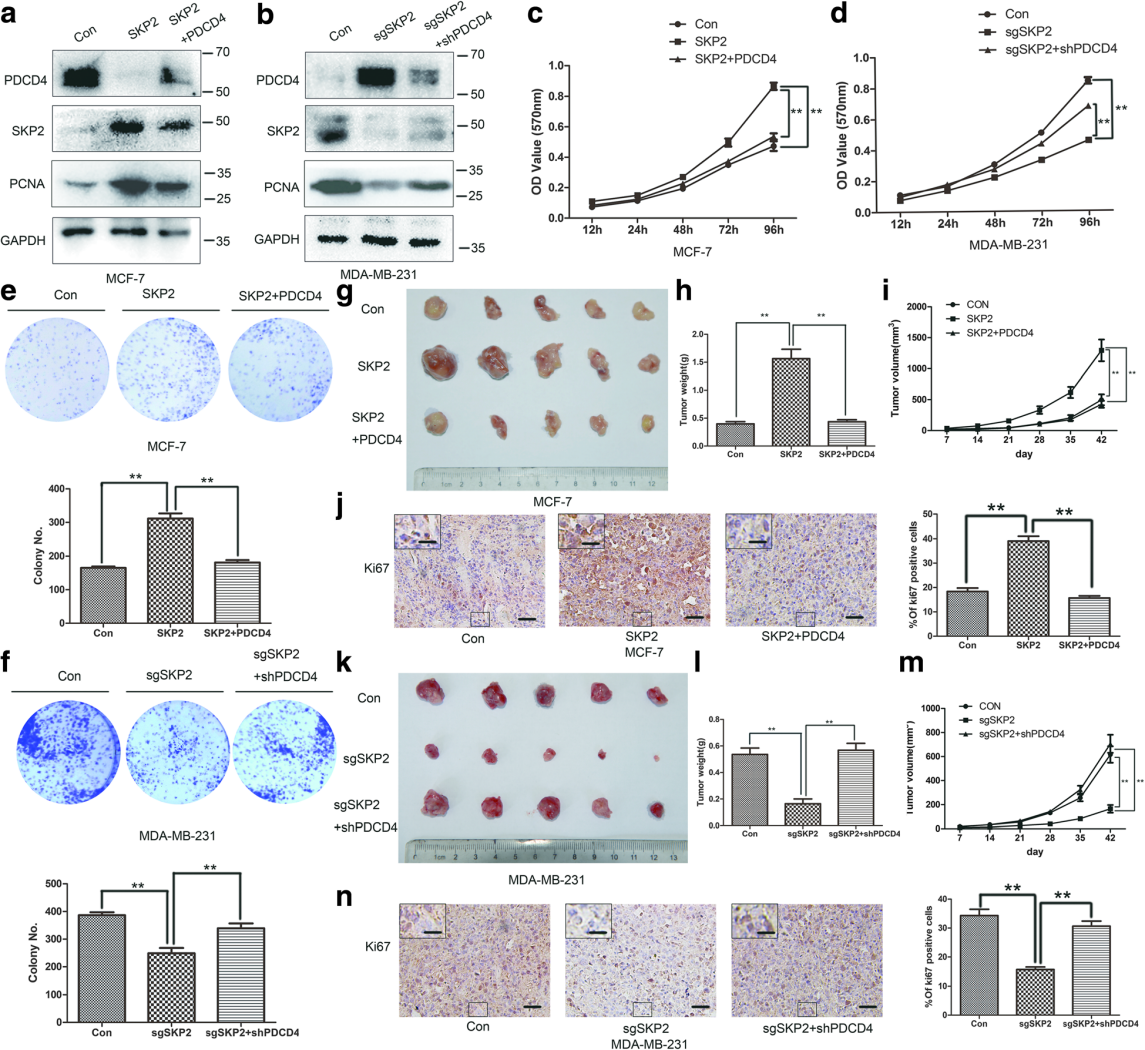
PDCD4 negatively regulate SKP2 protein levels and SKP2 increases the survival of breast cancer cells via PDCD4 suppression. **a** MCF7-Con and MCF-7-SKP2 cells were transfected with control or Flag-PDCD4 plasmid, selected and harvested for IB. **b** MDA-MB-231-Con and MDA-MB-231-sgSKP2 cells were transfected with control or shPDCD4 plasmid, selected and harvested for IB. **c**, **e** MCF-7-Con, MCF-7-SKP2 and MCF-7-SKP2 with PDCD4 stable expression cells were untreated or treated with radiation (6GY), followed by MTT assay and clonogenic survival assay (*n* = 3). **d**, **f** MDA-MB-231-Con, MDA-MB-231-sgSKP2 and MDA-MB-231-sgSKP2 with shPDCD4 stable expression cells were treated with radiation (6GY), followed by MTT assay and clonogenic survival assay (*n* = 3). **g** MCF-7-Con, MCF-7-SKP2 and MCF-7-SKP2 with PDCD4 stable expression cells were subcutaneously injected into nude mice (*n* = 5 for each group). A photo of five tumors aligned together were presented. **h** Tumor weight was measured. **i** Tumor size was monitored and calculated by caliper for up to 6 weeks. **j** Breast tumors were harvested from nude mice at 6 week for Ki-67 staining by IHC and quantitated (Scale bars, 50 um, Scale bars inside the box, 20 um). **k** MDA-MB-231-Con, MDA-MB-231-sgSKP2 and MDA-MB-231-sgSKP2 with shPDCD4 stable expression cells were subcutaneously injected into nude mice (n = 5 for each group). A photo of five tumors aligned together were presented. **l** Tumor weight was measured. **m** Tumor size was monitored and calculated by caliper for up to 6 weeks. **n** Breast tumors were harvested from nude mice at 6 week for Ki-67 staining by IHC and quantitated (Scale bars, 50 um, Scale bars inside the box, 20 um). **c-f, h-j, l-n** Data represent the mean ± SEM of three independent experiments. Student’s t-test used: *P < 0.05; **P < 0.01

Above research showed SKP2 negatively regulated PDCD4 expression promoted PDCD4 ubiquitination and degradation, we probed whether SKP2 promotes tumorigenesis via PDCD4 suppression in vitro and in vivo. To achieve this goal, we overexpressed PDCD4 in MCF-7-SKP2 cells and knocked down PDCD4 in MDA-MB-231-sgSKP2 cells. Western blot showed PDCD4 negatively regulated SKP2 protein levels (Fig. 3a, b). The same results were also observed in WT and SKP2^−/−^ MEF cells (Additional file 2: Figure S2b).

We then explored whether SKP2 promotes breast cancer cells proliferation via PDCD4 suppression. Western blot results showed SKP2 overexpression promoted PCNA protein expression in MCF-7 cells, while PDCD4 overexpression reversed the effect of SKP2 overexpression. On the other hand, SKP2 knockdown inhibited PCNA protein expression in MDA-MB-231 cells, while PDCD4 knockdown reversed the effect of SKP2 knockdown. These results imply SKP2 promotes cell proliferation via degrading PDCD4 in breast cancer. The cell survival efficiency was determined by colony-forming assay and MTT assay in MCF-7-vector, MCF-7-SKP2 and MCF-7-SKP2-PDCD4 cells as well as MDA-MB-231-vector, MDA-MB-231-sgSKP2 and MDA-MB-231-sgSKP2-sgPDCD4 cells. SKP2 overexpressed MCF-7 cells exhibited higher cell proliferation and colony formation compared with control cells, and PDCD4 overexpression reversed the effect of SKP2-MCF7 cells strikingly (Fig. 3c, e). On the other hand, SKP2 knockdown MDA-MB-231 cells exhibited lower cell proliferation and colony formation compared with control cells, and PDCD4 knockdown reversed the effect of MDA-MB-231-sgSKP2 cells (Fig. 3d, f). In vivo nude mice models also had similar results (Fig. 3g-i, k-m). IHC staining in breast tumors revealed that SKP2 overexpression promoted breast cancer cell proliferation in vivo, as determined by Ki-67 staining, and such promotion effect could be rescued by PDCD4 overexpression (Fig. 3j). On the other hand, Ki-67 staining showed SKP2 knockdown reduced breast cancer cell proliferation in vivo and such suppression effect could be rescued by PDCD4 knockdown (Fig. 3n). SKP2 and PDCD4 expression in nude mice was detected by immunohistochemical staining (Additional file 3: Figure S3). Altogether, these results suggest that SKP2 regulates breast cancer cells proliferation and breast cancer development via inhibiting PDCD4 expression.

### SKP2 increases the survival of breast cancer after radiation treatment via PDCD4 suppression

**Fig. 4:**
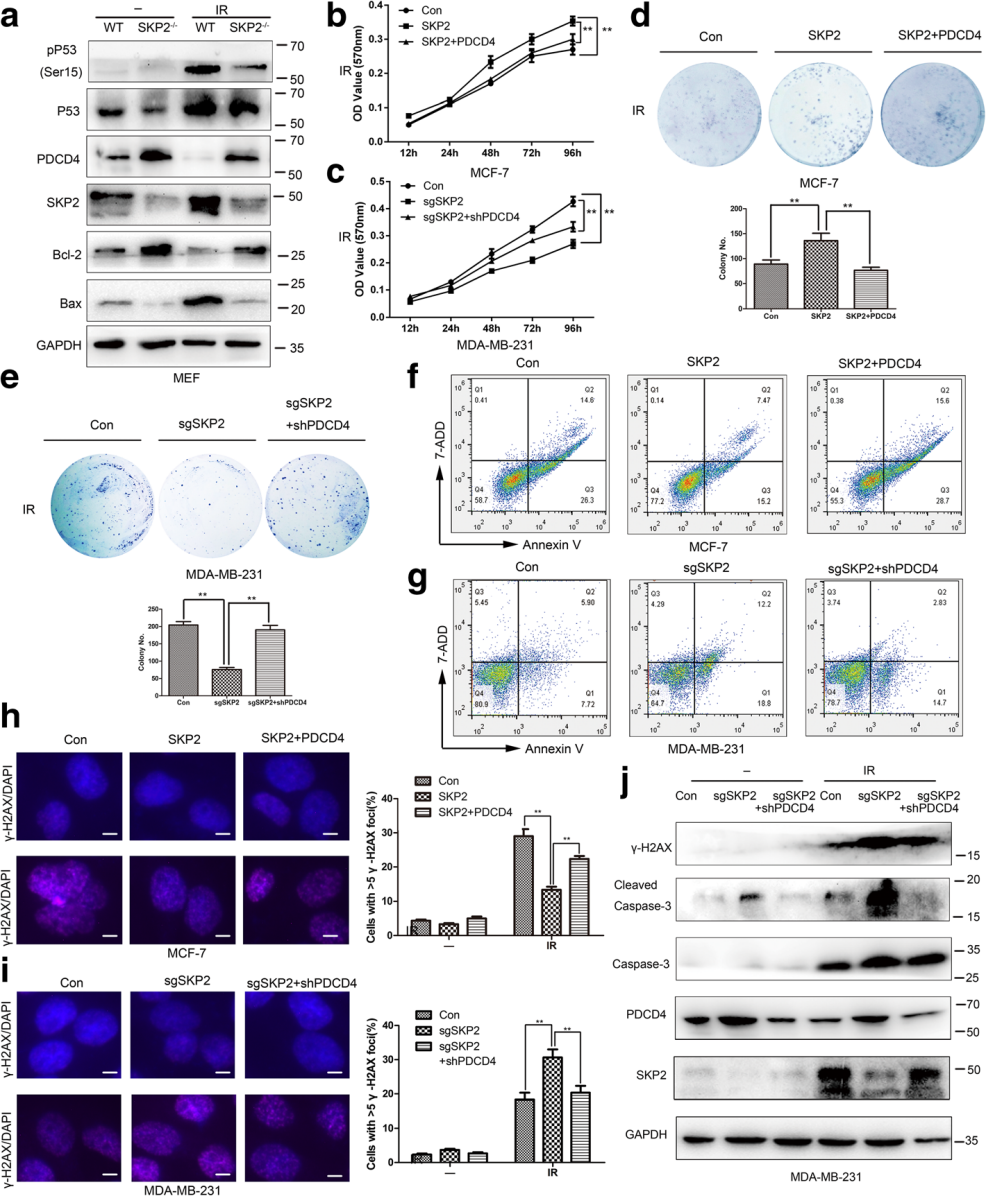
SCF^SKP2^ increases the survival of breast cancer after radiation treatment via PDCD4 suppression. **a** WT and SKP2^−/−^ MEF cells were treated with radiation (6GY), and after 1 h total lysates were prepared and subjected to IB analysis. **b**, **c** MCF-7-Con, MCF-7-SKP2 and MCF-7-SKP2 with PDCD4 stable expression cells were untreated or treated with radiation (6GY), followed by clonogenic survival assay and MTT assay (*n* = 3). **d**, **e** MDA-MB-231-Con, MDA-MB-231-sgSKP2 and MDA-MB-231-sgSKP2 with shPDCD4 stable expression cells were treated with radiation (6GY), followed by clonogenic survival assay and MTT assay (n = 3). **f** MCF-7-Con, MCF-7-SKP2 and MCF-7-SKP2 with PDCD4 stable expression cells were treated with radiation (6GY), and after 24 h induction of apoptosis was determined Annexin V staining. Annexin V-positive cells were analyzed with FACS, and a percent of Annexin V-positive cells was quantified and shown in the four-quadrant diagram. **g** MDA-MB-231-Con, MDA-MB-231-sgSKP2 and MDA-MB-231-sgSKP2 with shPDCD4 stable expression cells were untreated or treated with radiation (6GY), and after 24 h induction of apoptosis was determined by Annexin V staining. Annexin V-positive cells were analyzed with FACS, and a percent of Annexin V-positive cells was quantified and shown in the four-quadrant diagram. **h** MCF-7-Con, MCF-7-SKP2 and MCF-7-SKP2 with PDCD4 stable expression cells were untreated or treated with radiation (6GY) after 1 h, γ-H2AX expression was determined by immunostaining (Scale bars, 5 um). **i** MDA-MB-231-Con, MDA-MB-231-sgSKP2 and MDA-MB-231-sgSKP2 with shPDCD4 stable expression cells were untreated or treated with radiation (6GY). After 1 h, γ-H2AX expression was determined by immunostaining (Scale bars, 5 um). **j** MDA-MB-231-Con, MDA-MB-231-sgSKP2 and MDA-MB-231-sgSKP2 with shPDCD4 stable expression cells were untreated or treated with radiation (6GY) after 1 h, and total lysates were prepared and subjected to IB analysis. **b-e, h-i** Data represent the mean ± SEM of three independent experiments. Student’s t-test used: *P < 0.05; **P < 0.01

Since PDCD4 induces apoptosis [31] and participates in DNA-damage response in a P53-dependent manner [20], we probed whether SKP2 could suppress cell apoptosis, promote DNA damage response and increase survival after radiation via PDCD4 suppression. Western bolt showed PDCD4 was upregulated in SKP2^−/−^ MEF cells compared with WT MEF cells after radiation (Fig. 4a). The same result was also verified in PDCD4 knockdown MDA-MB-231 cells (Additional file 4: Figure S4a, b). Cell apoptosis marker Bax and DNA damage response marker pP53 (Ser15) were downregulated while Bcl-2 was upregulated in SKP2^−/−^ MEF cells compared with WT MEF cells after radiation. These results imply SKP2 could suppress cell apoptosis and promote DNA damage response via degrading PDCD4 in breast cancer. The cell survival efficiency was determined by colony-forming assay and MTT assay in MCF-7-vector, MCF-7-SKP2 and MCF-7-SKP2-PDCD4 cells after radiation treatment. The results showed SKP2-overexpressed MCF-7 cells exhibited higher cell proliferation and colony formation compared with control cells, and PDCD4 overexpression reversed the effect of SKP2-MCF7 cells (Fig. 4b, d). MDA-MB-231-vector, MDA-MB-231-sgSKP2 and MDA-MB-231-sgSKP2-sgPDCD4 cells verified the effects from the opposite side (Fig. 4c, e). Annexin V analysis measured by FACS showed increased expression of SKP2 reduced MCF-7 cell apoptosis, and PDCD4 transfection could significantly reverse SKP2 effects after radiation (Fig. 4f). On the contray, reducing the expression of SKP2 increased cell apoptosis as compared with MDA-MB-231-Con cells after radiation. PDCD4 knockdown could significantly reverse increased cell apoptosis (Fig. 4g). The results were verified also in MDA-MB-231 cell after radiation by Hoechst 33342 staining (Additional file 5: Figure S5). The immunofluorescence analysis revealed less γ-H2AX foci was noted in the nuclei of MCF-7-SKP2 cells than MCF-7 control cells after radiation, and PDCD4 transfection reversed the effect (*P* < 0.01) (Fig. 4h). More γ-H2AX foci was localised in the nuclei of MDA-MB-231-sgSKP2 cells than in control cells after radiation, and PDCD4 knockdown reversed the effect (*P* < 0.01) (Fig. 4i). The same effect was also verified on the levels of Cleaved-Caspase3 and γ-H2AX in MDA-MB-231 cells by using the western blot (Fig. 4j). These results suggest that SKP2 significantly inhibits cell apoptosis and promotes DNA-damage response via PDCD4 suppression after radiation in breast cancer cells.

### SKP2 and PDCD4 expression in human tissues and clinical correlation

**Fig. 5:**
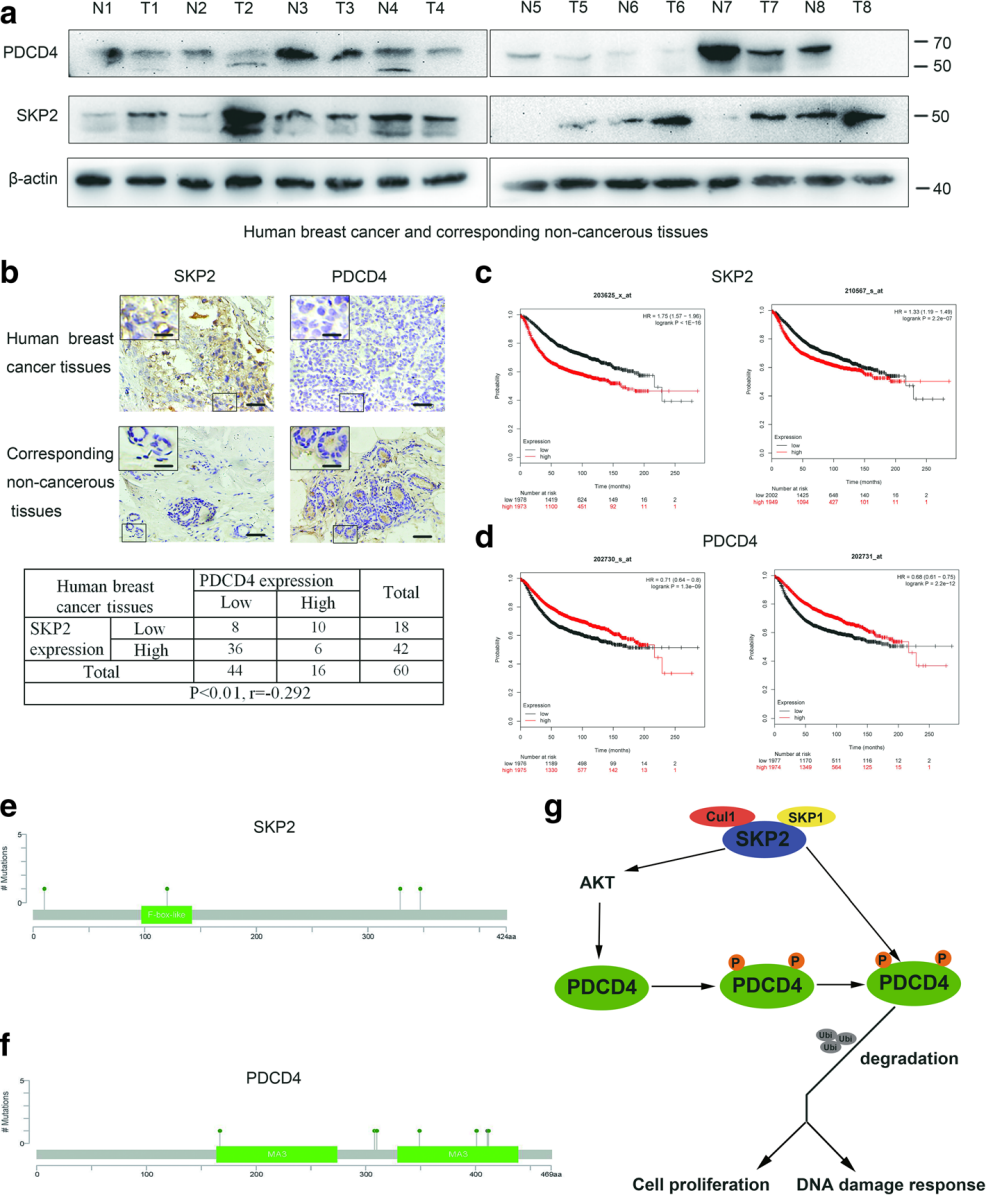
SKP2 and PDCD4 expression in human tissues and clinical correlation. **a** Human breast cancer and corresponding non-cancerous tissues were used for SKP2 and PDCD4 expression by western blot. **b** Human breast cancer and corresponding non-cancerous tissues were used for SKP2 and PDCD4 staining by IHC and quantitated (Scale bars, 50 um, Scale bars inside the box, 20 um). Chi-square test was used to analyze the correlation between SKP2 and PDCD4. P < 0.05 was considered as significant. **c** SKP2 overexpression was significantly associated with poor prognosis in human breast cancer patients (*P* < 0.001). Gene expression data, relapse free and overall survival information were downloaded from GEO (Affymetrix microarrays only), EGA and TCGA. The database was handled by a PostgreSQL server, which integrates gene expression and clinical data simultaneously. To analyze the prognostic value of a particular gene, the patient samples were split into two groups according to various quantile expressions of the proposed biomarker. Cut-off value for survival was determined with ROC curve. The two patient cohorts were compared by a Kaplan-Meier survival plot, and the hazard ratio with 95% confidence intervals and logrank *P* value were calculated. **d** PDCD4 overexpression were significantly associated with favourable prognosis in human breast cancer patients (P < 0.001). **e, f** Mutation detection of SKP2 and PDCD4 in human breast cancer patients were perform in the breast cancer patients database of cBioPortal for cancer Genomics. **g** The working model of SKP2 via PDCD4 in tumorigenesis and DNA-damage response

To understand the correlation of SKP2 and PDCD4, we performed Western blot and IHC to determine the expression of these proteins in human breast cancer samples and para-carcinoma samples (Fig. 5a, b). SKP2 was upregulated and PDCD4 was downregulated in breast cancer samples. SKP2 expression levels showed negative correlation with PDCD4 expression levels. Next, we studied the relationship between mRNA expression of SKP2 or PDCD4 and clinical outcome using a Kaplan–Meier plotter (http://www.kmplot.com) [31]. SKP2 mRNA high expression was found to be correlated to significantly poor prognosis for breast cancer patients (Affymetrix ID: 203625_x_at, HR = 1.75 (1.57–1.96), *p* < 1e-16; Affymetrix ID: 203567_x_at, HR = 1.33 (1.19–1.49), *p* = 2.2e - 07) (Fig. 5c). On the contrary, PDCD4 mRNA high expression was significantly associated with favourable prognosis (Affymetrix ID: 202730_x_at, HR = 0.71 (0.64–0.8), *p* = 1.3e - 09; Affymetrix ID: 202731_x_at, HR = 0.68 (0.61–0.75), p = 2.2e - 12) (Fig. 5d). Mutation detection of breast cancer patients in cBioPortal for Cancer Genomics showed SKP2 mutated at the F-box domain and PDCD4 mutated at the MA-3 domain [32], which is the eIF4A binding domain [33] (Fig. 5e, f). These results together with our findings suggest that the SKP2-PDCD4 axis plays an important role in breast cancer development and represents an important biomarker for survival outcome of breast cancer patients (Fig. 5g).

### SKP2 inhibitor SMIP004 increases the effect of tumor radiotherapy

**Fig. 6:**
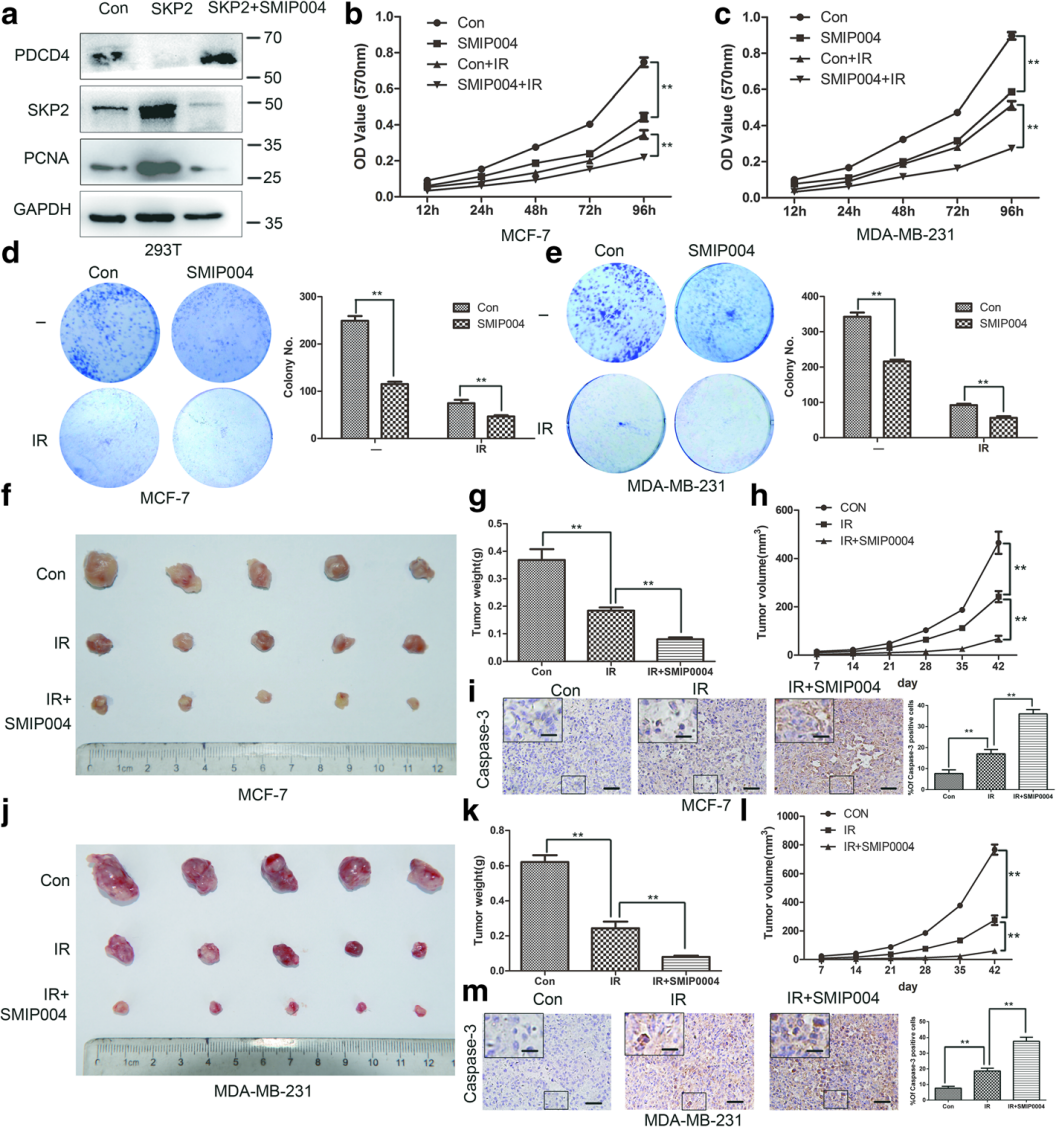
SKP2 inhibitor SMIP004 increases the effect of tumor radiotherapy. **a** SMIP004 downregulated SKP2 expression levels and upregulated PDCD4 expression levels. 293 T cells were transfected with Flag-SKP2 and control plasmid for 48 h, then untreated or treated with SMIP004(40 μM) for 24 h and harvested for IB. **b**, **c** MCF-7 or MDA-MB-231 were treated or untreated with SMIP004 (40 μM) for 24 h, then untreated or treated with radiation (6GY), followed by MTT assay (n = 3). **d, e** MCF-7 or MDA-MB-231 were treated or untreated with SMIP004 (40 μM) for 24 h, then untreated or treated with radiation (6GY), followed by clonogenic survival assay (n = 3). **f, j** MCF-7 or MDA-MB-231 cells were subcutaneously injected into nude mice (*n* = 5 for each group), then untreated or treated with radiation at 0.1GY/min for 10 min twice a week from 4 to 6 week or radiation at 0.1GY/min for 10 min and SMIP004 (50 mg/kg) twice a week from 4 to 6 week. A photo of five tumors aligned together were presented. **g, k ** Tumor weight was measured. **h, l** Tumor size was monitored and calculated by caliper for up to 6 weeks (see Methods). **i, m** Breast tumors were harvested from nude mice at 6 week for Caspase-3 staining by IHC and quantitated (Scale bars, 50 um, Scale bars inside the box, 20 um). **b-e, g-i, k-m** Data represent the mean ± SEM of three independent experiments. Student’s t-test used: *P < 0.05; **P < 0.01

The above research results indicate that SKP2 participates in DNA-damage response and cell survival after radiation, we further investigated whether SKP2 inhibitors could be used as potential radiosensitizers for treating breast cancer. We used SMIP004, which was found to downregulate SKP2 and stabilise p27 [34], to prove our concept. Western blot analysis showed SMIP004 significantly downregulated SKP2 expression levels and upregulated PDCD4 expression levels (Fig. 6a). SMIP004 inhibited PCNA protein expression while PDCD4 knockdown reversed the effect of SMIP004 (Fig. 6a). MCF-7 or MDA-MB-231 cells treated with SMIP004 exhibited lower cell proliferation and colony formation compared with control cells after radiation treatment (Fig. 6b-e). Immunofluorescence showed moreγ-H2AX foci localised in the nuclei of MCF-7 or MDA-MB-231 cells treated with SMIP004 than cells after radiation treatment (Additional file 6: Figure S6a, b). The inhibitory effects of SMIP004 combine with radiation treatment were also observed in vivo nude mice models (Fig. 6f-h, j-l). Caspase-3 and γ-H2AX staining showed SMIP004 promoted breast cancer cells apoptosis and increased DNA damage in vivo after radiation (Fig. 6i, m, Additional file 7: Figure S7a, b). These results showed radiotherapy combined with SMIP004 may have satisfactory clinical effects on breast cancer patients. In conclusion, SKP2 inhibitor can be used as a novel radiosensitizer in breast cancer clinical trials.

## Discussion

SKP2 is a major component of the SCF^SKP2^ E3 complex which catalysing the ubiquitination of proteins. This complex promotes the ubiquitination of cell cycle proteins, including P27 [28], P21 [35], P57 [36], cyclin A [37], cyclin E [37], cyclin D1 [38] and tumor suppressor proteins, including BRCA2 [39], SMAD4 [40], RASSF1A [41], FOXO1 [42] and so on. PDCD4 is a tumor suppressor that inhibits the formation of pre-initiation complexes by combining with eIF4A [19]. PDCD4 regulates cellular DNA-damage response by inhibiting the translation process of P53 [20]. Our study showed PDCD4 is a novel ubiquitination substrate of SKP2, which helps to clarify SKP2 tumor promotion and DNA damage response action.

Our study has revealed several significant findings related to clinical applications. First, our study provides a new path of SKP2 promoting tumorigenesis and in response to DNA-damage through PDCD4 degradation. We unequivocally show that SCF^SKP2^ is an E3 ligase for PDCD4, which triggers K48-linked ubiquitination and degradation of PDCD4, in turn causing enhanced cell proliferation, decreased cell apoptosis and enhanced DNA-damage response. PDCD4 also negatively regulates SKP2 expression. Our data provides a new approach to inhibit cell proliferation and increase radiosensitivity after radiation by SKP2 targeting.

Second, as SKP2 expression was negatively associated with PDCD4 (*P* < 0.01) in clinical samples, the deregulation of PDCD4 may represent an oncogenic event. This result also implicates that SKP2 overexpression may contribute to breast cancer pathogenesis by suppressing PDCD4. PDCD4 directly inhibits the translation of P53 protein [20]. 5’-UTR of P53 mRNA is a stable loop stem structure and affects P53 protein translation [43]. The RNA-binding domain of the PDCD4 protein can specifically combine with P53 5’-UTR and inhibit the translation of P53 [20]. Due to the effect of PDCD4 on P53, a variety of biological processes of cells, including cell growth and radiation or pharmacy resistance will undergo abnormal changes. This notion is indeed supported by our results. Our results showed SKP2 promotes DNA damage response and radiation tolerance via inhibiting PDCD4 expression. PDCD4 silencing rescues the effects of SKP2 after radiation. Moreover, since the levels of PDCD4 expression in tumor cells are closely related to the sensitivity of antitumor drugs. Upregulation of PDCD4 expression in tumor cells increases the sensitivity for certain anti-cancer drugs, while downregulation of PDCD4 expression reduces the sensitivity for certain anti-cancer drugs [44]. Previous research together with our results imply that SKP2 is potential radiotherapy or anti-tumor drug sensitization target.

Third, we verified SKP2 inhibitor could improve the efficacy of radiotherapy. SMIP004 is a novel cancer cell selective apoptosis inducer of human prostate cancer cells. It was found to downregulate SKP2 and to stabilise p27 [34]. Our results showed SMIP004 combined with radiation had a remarkable effect in human breast cancer cells. SMIP004 increased breast cancer cell radiation sensitivity. Radiation with SMIP004 induced more apoptosis than radiation alone in vitro. Human breast cancer xenografts in mouse models also showed the same result. SMIP004 may be a sensitizer for radiotherapy of breast cancer.

In addition, we also found increased phosphorylated DNA-PK protein levels in SKP2 overexpressed breast cancer cells compared with control cells after radiation, implying that SKP2 may also participate in DNA-damage repair by involving in non-homologous end joining (NHEJ) [45]. DNA-PK is required for the NHEJ pathway of DNA repair, which rejoins DSBs [46]. It is also required for V (D) J recombination [47]. Moreover, SCF^SKP2^ has been proven to affect V (D) J recombination by restricting accumulation of RAG-2 to the G1 phase, at which time NHEJ is the prevalent form of DNA repair. SCF^SKP2^ may coordinate the generation of RAG-induced DNA breaks with their repair by NHEJ [48]. The results hint that SKP2 may play an important role in DNA-damage repair and NHEJ.

## Conclusions

In conclusions, our study uncovers a molecular mechanism of SKP2 controlling PDCD4 stability by mediating PDCD4 ubiquitination and degradation. We identify the SKP2–PDCD4 axis as a key pathway for tumorigenesis, cell apoptosis and DNA-damage response. SKP2 inhibitor SMIP004 combine with radiation treatment showed remarkable inhibitory effects on breast cancer cells. Given these results, SKP2-targeted drugs may act as sensitizers for the radiotherapy of breast cancer.

## Additional files

Additional file 1: **Figure S1.** SKP2ΔF can’t bind to PDCD4 or regulate PDCD4 protein levels. (a) 293 T cells were transfected with indicated vector, SKP2, SKP2△F and SKP2△N plasmids and harvested for immunoprecipitation assay. (b) Overexpression of SKP2ΔF has no effect on the levels of PDCD4 protein in MCF-7 cells: MCF-7 cells were transfected with SKP2 or vector control, followed by IB for protein expression. (TIF 6679 kb)

Additional file 2: **Figure S2.** SKP2 and PDCD4 showed negative correlation and PDCD4 retroregulates SKP2 expression in MEF cells. (a) SKP2^−/−^ and WT mouse tissues were used for SKP2, PDCD4 staining by IHC and quantitated (Scale bars, 50 um, Scale bars inside the box, 20 um). Data represent the mean ± SEM of three independent experiments. Student’s t-test used: *P < 0.05; **P < 0.01. (b) Primary WT and SKP2^−/−^ MEFs were transfected with shRNAs (shLuc) or PDCD4 shRNAs (shPDCD4), selected and harvested for IB. (TIF 13811 kb)

Additional file 3: **Figure S3.** SKP2 and PDCD4 expression in breast tumors from nude mice were detected by immunohistochemical staining. (a) Breast tumors from MCF-7-Con, MCF-7-SKP2 and MCF-7-SKP2 with PDCD4 stable expression cells were harvested from nude mice at 6 weeks for SKP2 and PDCD4 staining by IHC and quantitated (Scale bars, 50 um, Scale bars inside the box, 20 um). Data represent the mean ± SEM of three independent experiments. Student’s t-test used: *P < 0.05; **P < 0.01. (b) Breast tumors from MDA-MB-231-Con, MDA-MB-231-sgSKP2 and MDA-MB-231-sgSKP2 with shPDCD4 stable expression cells were harvested from nude mice at 6 week for SKP2 and PDCD4 staining by IHC and quantitated (Scale bars, 50 um, Scale bars inside the box, 20 um). Data represent the mean ± SEM of three independent experiments. Student’s t-test used: *P < 0.05; **P < 0.01. (TIF 31660 kb)

Additional file 4: **Figure S4.** PDCD4 is upregulated in MDA-MB-231-sgSKP2 cells after radiation. (a) SKP2 expression of MDA-MB-231-Con and MDA-MB-231-sgSKP2 cells before and after radiation is determined by immunostaining (Scale bars, 25 um). (b) PDCD4 expression of MDA-MB-231-Con and MDA-MB-231-sgSKP2 cells before and after radiation is determined by immunostaining (Scale bars, 25 um). (TIF 39287 kb)

Additional file 5: **Figure S5.** SKP2 promotes cell apoptosis by inhibiting PDCD4 in MDA-MB-231 cells. Induction of apoptosis was determined by by Hoechst 33342 staining after 24 h cells were treated radiation (6GY) in MDA-MB-231-Con, MDA-MB-231-sgSKP2 and MDA-MB-231-sgSKP2 with shPDCD4 stable expression cells (Scale bars, 50 um). Data represent the mean ± SEM of three independent experiments. Student’s t-test used: *P < 0.05; **P < 0.01. (TIF 21910 kb)

Additional file 6: **Figure S6.** SMIP004 promotes breast cancer cell apoptosis after radiation. (a, b) MCF-7 or MDA-MB-231 cells were untreated or treated with SMIP004 (40 μM) for 24 h, then untreated or treated with radiation (6GY). After 1 h, γ-H2AX expression is determined by immunostaining. DSBs were determined by analysis of γ-H2AX by immunostaining (Scale bars, 5 um). Data represent the mean ± SEM of three independent experiments. Student’s t-test used: *P < 0.05; **P < 0.01. (TIF 10302 kb)

Additional file 7: **Figure S7.** γ-H2AX expression in breast tumors from nude mice were detected by immunohistochemical staining. (a) Breast tumors from MCF-7 cells treated or untreated with SMIP004 or radiation were harvested from nude mice at 6 week for γ-H2AX staining by IHC and quantitated (Scale bars, 50 um, Scale bars inside the box, 20 um). Data represent the mean ± SEM of three independent experiments. Student’s t-test used: *P < 0.05; **P < 0.01. (b) Breast tumors from MDA-MB-231 cells treated or untreated with SMIP004 or radiation were harvested from nude mice at 6 week for γ-H2AX staining by IHC and quantitated (Scale bars, 50 um, Scale bars inside the box, 20 um). Data represent the mean ± SEM of three independent experiments. Student’s t-test used: *P < 0.05; **P < 0.01. (TIF 17751 kb)

## Abbreviations

AKT: Protein kinase B
ATM: Ataxia telangiectasia-mutated gene
BRCA2: Breast cancer susceptibility gene2
Co-IP: Co-immunoprecipitation
DSBs: DNA double—strand breaks
eIF4A: Eukaryotic initiation factor-4A
eIF4G: Eukaryotic initiation factor-4G
FOXO1: Forkhead box O1
HR: Homologous recombination
MEF: Mouse embryonic fibroblast
MRN: MRE11/RAD50/NBS1 complex
NHEJ: Non-homologous end joining
PDCD4: Programmed cell death protein 4
PI: Propidine iodide
RAG-2: Recombination-activating gene2
RASSF1A: Ras association domain-containing protein 1A
SCF: Skp, Cullin, F-box containing complex
SKP2: S-phase kinase-associated protein 2
SMAD4: SMAD family member 4
UTR: Untranslated Region
V (D) J: Variable (diversity) joining
β-TRCP: Beta-transducin repeats containing proteins

## References

[1] DeSantis C, Ma J, Bryan L, Jemal A (2014). Breast cancer statistics, 2013. CA Cancer J Clin.

[2] Bellon JR, Harris EE, Arthur DW, Bailey L, Carey L, Goyal S, Halyard MY, Horst KC, Moran MS, MacDonald SM (2011). ACR appropriateness criteria(R) conservative surgery and radiation--stage I and II breast carcinoma: expert panel on radiation oncology: breast. Breast J.

[3] Darby S, McGale P, Correa C, Taylor C, Arriagada R, Clarke M, Cutter D, Davies C, Ewertz M, Early Breast Cancer Trialists' Collaborative G (2011). Effect of radiotherapy after breast-conserving surgery on 10-year recurrence and 15-year breast cancer death: meta-analysis of individual patient data for 10,801 women in 17 randomised trials. Lancet.

[4] Bai C, Sen P, Hofmann K, Ma L, Goebl M, Harper JW, Elledge SJ (1996). SKP1 connects cell cycle regulators to the ubiquitin proteolysis machinery through a novel motif, the F-box. Cell.

[5] Craig KL, Tyers M (1999). The F-box: a new motif for ubiquitin dependent proteolysis in cell cycle regulation and signal transduction. Prog Biophys Mol Biol.

[6] Nakayama KI, Nakayama K (2005). Regulation of the cell cycle by SCF-type ubiquitin ligases. Semin Cell Dev Biol.

[7] Wang HB, Cui JH, Bauzon F, Zhu L (2010). A comparison between Skp2 and FOXO1 for their cytoplasmic localization by Akt1. Cell Cycle.

[8] Seki R, Okamura T, Koga H, Yakushiji K, Hashiguchi M, Yoshimoto K, Ogata H, Imamura R, Nakashima Y, Kage M (2003). Prognostic significance of the F-box protein Skp2 expression in diffuse large B-cell lymphoma. Am J Hematol.

[9] Wang ZW, Gao DM, Fukushima H, Inuzuka H, Liu PD, Wan LX, Sarkar FH, Wei WY (2012). Skp2: a novel potential therapeutic target for prostate cancer. Biochim Et Biophys Acta Rev Cancer.

[10] Rose AE, Wang GM, Hanniford D, Monni S, Tu T, Shapiro RL, Berman RS, Pavlick AC, Pagano M, Darvishian F (2011). Clinical relevance of SKP2 alterations in metastatic melanoma. Pigment Cell Melanoma Res.

[11] Fang FM, Chien CY, Li CF, Shiu WY, Chen CH, Huang HY (2009). Effect of S-phase kinase-associated protein 2 expression on distant metastasis and survival in nasopharyngeal carcinoma patients. Int J Radiat Oncol Biol Phys.

[12] Radke S, Pirkmaier A, Germain D (2005). Differential expression of the F-box proteins Skp2 and Skp2B in breast cancer. Oncogene.

[13] Wu J, Zhang X, Zhang L, Wu CY, Rezaeian AH, Chan CH, Li JM, Wang J, Gao Y, Han F (2012). Skp2 E3 ligase integrates ATM activation and homologous recombination repair by Ubiquitinating NBS1. Mol Cell.

[14] Chen Y, Knosel T, Kristiansen G, Pietas A, Garber ME, Matsuhashi S, Ozaki I, Petersen I (2003). Loss of PDCD4 expression in human lung cancer correlates with tumour progression and prognosis. J Pathol.

[15] Wang Q, Sun Z, Yang HS (2008). Downregulation of tumor suppressor Pdcd4 promotes invasion and activates both beta-catenin/Tcf and AP-1-dependent transcription in colon carcinoma cells. Oncogene.

[16] Gao F, Zhang P, Zhou CX, Li JF, Wang Q, Zhu FL, Ma CH, Sun WS, Zhang LN (2007). Frequent loss of PDCD4 expression in human glioma: possible role in the tumorigenesis of glioma. Oncol Rep.

[17] Zhang H, OzakiZ I, Mizuta T, Hamajima H, Yasutake T, Eguchi Y, Ideguchi H, Yamamoto K, Matsuhashi S (2006). Involvement of programmed cell death 4 in transforming growth factor-beta 1-induced apoptosis in human hepatocellular carcinoma. Oncogene.

[18] Yang HS, Jansen AP, Komar AA, Zheng XJ, Merrick WC, Costes S, Lockett SJ, Sonenberg N, Colburn NH (2003). The transformation suppressor Pdcd4 is a novel eukaryotic translation initiation factor 4A binding protein that inhibits translation. Mol Cell Biol.

[19] Waters LC, Strong SL, Ferlemann E, Oka O, Muskett FW, Veverka V, Banerjee S, Schmedt T, Henry AJ, Klempnauer KH, Carr MD (2011). Structure of the tandem MA-3 region of Pdcd4 protein and characterization of its interactions with eIF4A and eIF4G molecular mechanisms of a tumor suppressor. J Biol Chem.

[20] Schmid T, Jansen AP, Baker AR, Hegamyer G, Hagan JP, Colburn NH (2008). Translation inhibitor Pdcd4 is targeted for degradation during tumor promotion. Cancer Res.

[21] Singh P, Marikkannu R, Bitomsky N, Klempnauer KH (2009). Disruption of the Pdcd4 tumor suppressor gene in chicken DT40 cells reveals its role in the DNA-damage response. Oncogene.

[22] Dorrello NV, Peschiaroli A, Guardavaccaro D, Colburn NH, Sherman NE, Pagano M (2006). S6K1- and beta TRCP-mediated degradation of PDCD4 promotes protein translation and cell growth. Science.

[23] Liang SM, Yao QM, Wei DY, Liu M, Geng F, Wang Q, Wang YS (2019). KDM6B promotes ovarian cancer cell migration and invasion by induced transforming growth factor-beta 1 expression. J Cell Biochem.

[24] Wang J, Han F, Wu J, Lee SW, Chan CH, Wu CY, Yang WL, Gao Y, Zhang X, Jeong YS (2011). The role of Skp2 in hematopoietic stem cell quiescence, pool size, and self-renewal. Blood.

[25] Tsvetkov LM, Yeh KH, Lee SJ, Sun H, Zhang H (1999). P27(Kip1) ubiquitination and degradation is regulated by the SCFSkp2 complex through phosphorylated Thr187 in p27. Curr Biol.

[26] Palamarchuk A, Efanov A, Maximov V, Aqeilan RI, Croce CM, Pekarsky Y (2005). Akt phosphorylates and regulates Pdcd4 tumor suppressor protein. Cancer Res.

[27] Chan CH, Li CF, Yang WL, Gao Y, Lee SW, Feng ZZ, Huang HY, Tsai KKC, Flores LG, Shao YP (2012). The Skp2-SCF E3 ligase regulates Akt ubiquitination, glycolysis, Herceptin sensitivity, and tumorigenesis. Cell.

[28] Carrano AC, Eytan E, Hershko A, Pagano M (1999). SKP2 is required for ubiquitin-mediated degradation of the CDK inhibitor p27. Nat Cell Biol.

[29] Huang H, Regan KM, Wang F, Wang D, Smith DI, van Deursen JM, Tindall DJ (2005). Skp2 inhibits FOXO1 in tumor suppression through ubiquitin-mediated degradation. Proc Natl Acad Sci U S A.

[30] Yang WL, Zhang X, Lin HK (2010). Emerging role of Lys-63 ubiquitination in protein kinase and phosphatase activation and cancer development. Oncogene.

[31] Gyorffy B, Lanczky A, Eklund AC, Denkert C, Budczies J, Li QY, Szallasi Z (2010). An online survival analysis tool to rapidly assess the effect of 22,277 genes on breast cancer prognosis using microarray data of 1,809 patients. Breast Cancer Res Treat.

[32] Cerami E, Gao J, Dogrusoz U, Gross BE, Sumer SO, Aksoy BA (2012). The cBio Cancer genomics portal: an open platform for exploring multidimensional Cancer genomics data (vol 2, pg 401, 2012). Cancer Discov.

[33] Lankat-Buttgereit B, Goke R (2009). The tumour suppressor Pdcd4: recent advances in the elucidation of function and regulation. Biol Cell.

[34] Rico-Bautista E, Yang CC, Lu L, Roth GP, Wolf DA (2010). Chemical genetics approach to restoring p27Kip1 reveals novel compounds with antiproliferative activity in prostate cancer cells. BMC Biol.

[35] Bornstein G, Bloom J, Sitry-Shevah D, Nakayama K, Pagano M, Hershko A (2003). Role of the SCFSkp2 ubiquitin ligase in the degradation of p21(Cip1) in S phase. J Biol Chem.

[36] Kamura T, Hara T, Kotoshiba S, Yada M, Ishida N, Imaki H, Hatakeyama S, Nakayama K, Nakayama KI (2003). Degradation of p57(Kip2) mediated by SCFSkp2 - dependent ubiquitylation. Proc Natl Acad Sci U S A.

[37] Nakayama K, Nagahama H, Minamishima YA, Matsumoto M, Nakamichi I, Kitagawa K, Shirane M, Tsunematsu R, Tsukiyama T, Ishida N (2000). Targeted disruption of Skp2 results in accumulation of cyclin E and p27(Kip1), polyploidy and centrosome overduplication. EMBO J.

[38] Yu ZK, Gervais JLM, Zhang H (1998). Human CUL-1 associates with the SKP1/SKP2 complex and regulates p21(CIP1/WAF1) and cyclin D proteins. Proc Natl Acad Sci U S A.

[39] Moro L, Arbini AA, Marra E, Greco M (2006). Up-regulation of Skp2 after prostate cancer cell adhesion to basement membranes results in BRCA2 degradation and cell proliferation. J Biol Chem.

[40] Liang M, Liang YY, Wrighton K, Ungermannova D, Wang XP, Brunicardi FC, Liu XD, Feng XH, Lin X (2004). Ubiquitination and proteolysis of cancer-derived Smad4 mutants by SCFSkp2. Mol Cell Biol.

[41] Song MS, Song SJ, Kim SJ, Nakayama K, Nakayama KI, Lim DS (2008). Skp2 regulates the antiproliferative function of the tumor suppressor RASSF1A via ubiquitin-mediated degradation at the G(1)-S transition. Oncogene.

[42] Huang H, Regan KM, Wang F, Wang DP, Smith DI, van Deursen JMA, Tindall DJ (2005). Skp2 inhibits FOX01 in tumor suppression through ubiquitin-mediated degradation. Proc Natl Acad Sci U S A.

[43] Yang DQ, Halaby MJ, Zhang Y (2006). The identification of an internal ribosomal entry site in the 5 '-untranslated region of p53 mRNA provides a novel mechanism for the regulation of its translation following DNA damage. Oncogene.

[44] Jansen AP, Camalier CE, Stark C, Colburn NH (2004). Characterization of programmed cell death 4 in multiple human cancers reveals a novel enhancer of drug sensitivity. Mol Cancer Ther.

[45] Burma S, Chen DJ (2004). Role of DNA-PK in the cellular response to DNA double-strand breaks. DNA Repair.

[46] Uematsu N, Weterings E, Yano K, Morotomi-Yano K, Jakob B, Taucher-Scholz G, Mari PO, van Gent DC, Chen BPC, Chen DJ (2007). Autophosphorylation of DNA-PKCS regulates its dynamics at DNA double-strand breaks. J Cell Biol.

[47] Ma YM, Schwarz K, Lieber MR (2005). The Artemis: DNA-PKcs endonuclease cleaves DNA loops, flaps, and gaps. DNA Repair.

[48] Jiang H, Chang FC, Ross AE, Lee JY, Nakayama K, Nakayama K, Desiderio S (2005). Ubiquitylation of RAG-2 by Skp2-SCF links destruction of the V(D)J recombinase to the cell cycle. Mol Cell.

