# Supplementary figures and images for: SKP2 promotes breast cancer tumorigenesis and radiation tolerance through PDCD4 ubiquitination
Source: J Exp Clin Cancer Res. 2019 Feb 13;38:76. doi: 10.1186/s13046-019-1069-3 (PMC6375223; doi:10.1186/s13046-019-1069-3)

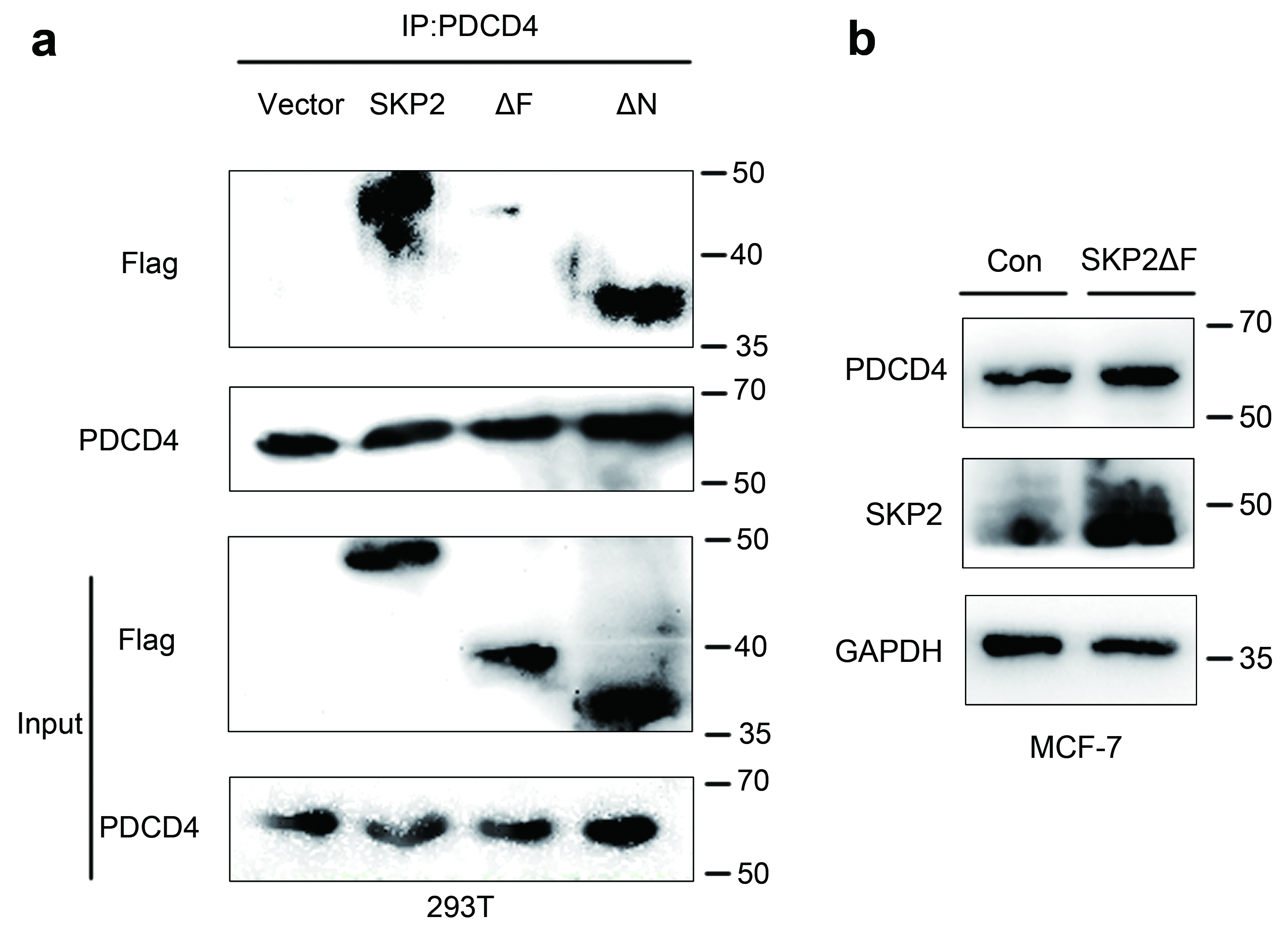

Supplement: Supplementary file 1 — Figure S1. SKP2ΔF can’t bind to PDCD4 or regulate PDCD4 protein levels. (a) 293 T cells were transfected with indicated vector, SKP2, SKP2△F and SKP2△N plasmids and harvested for immunoprecipitation assay. (b) Overexpression of SKP2ΔF has no effect on the levels of PDCD4 protein in MCF-7 cells: MCF-7 cells were transfected with SKP2 or vector control, followed by IB for protein expression. (TIF 6679 kb) [file 13046_2019_1069_MOESM1_ESM.tif]

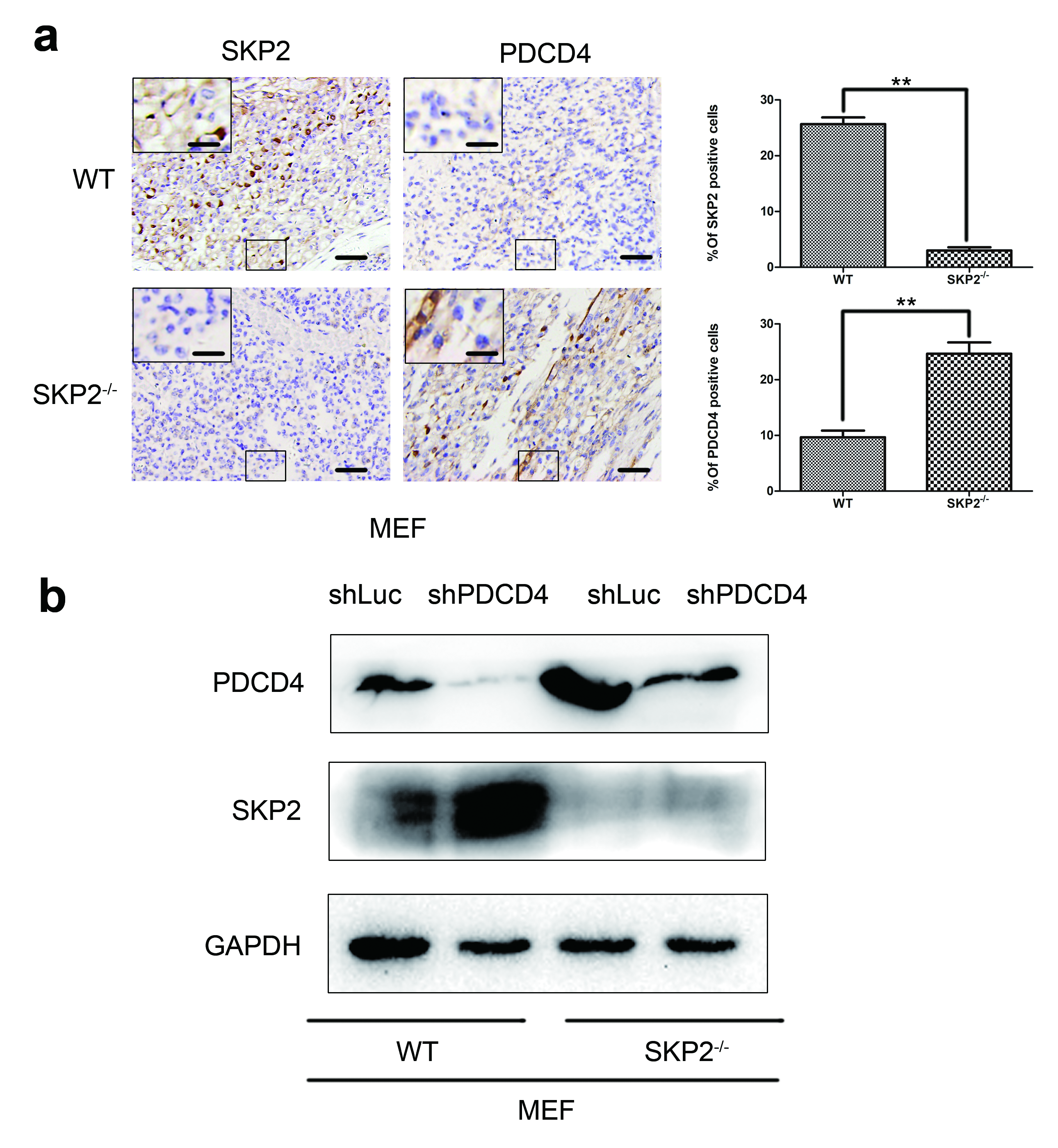

Supplement: Supplementary file 2 — Figure S2. SKP2 and PDCD4 showed negative correlation and PDCD4 retroregulates SKP2 expression in MEF cells. (a) SKP2−/− and WT mouse tissues were used for SKP2, PDCD4 staining by IHC and quantitated (Scale bars, 50 um, Scale bars inside the box, 20 um). Data represent the mean ± SEM of three independent experiments. Student’s t-test used: *P < 0.05; **P < 0.01. (b) Primary WT and SKP2−/− MEFs were transfected with shRNAs (shLuc) or PDCD4 shRNAs (shPDCD4), selected and harvested for IB. (TIF 13811 kb) [file 13046_2019_1069_MOESM2_ESM.tif]

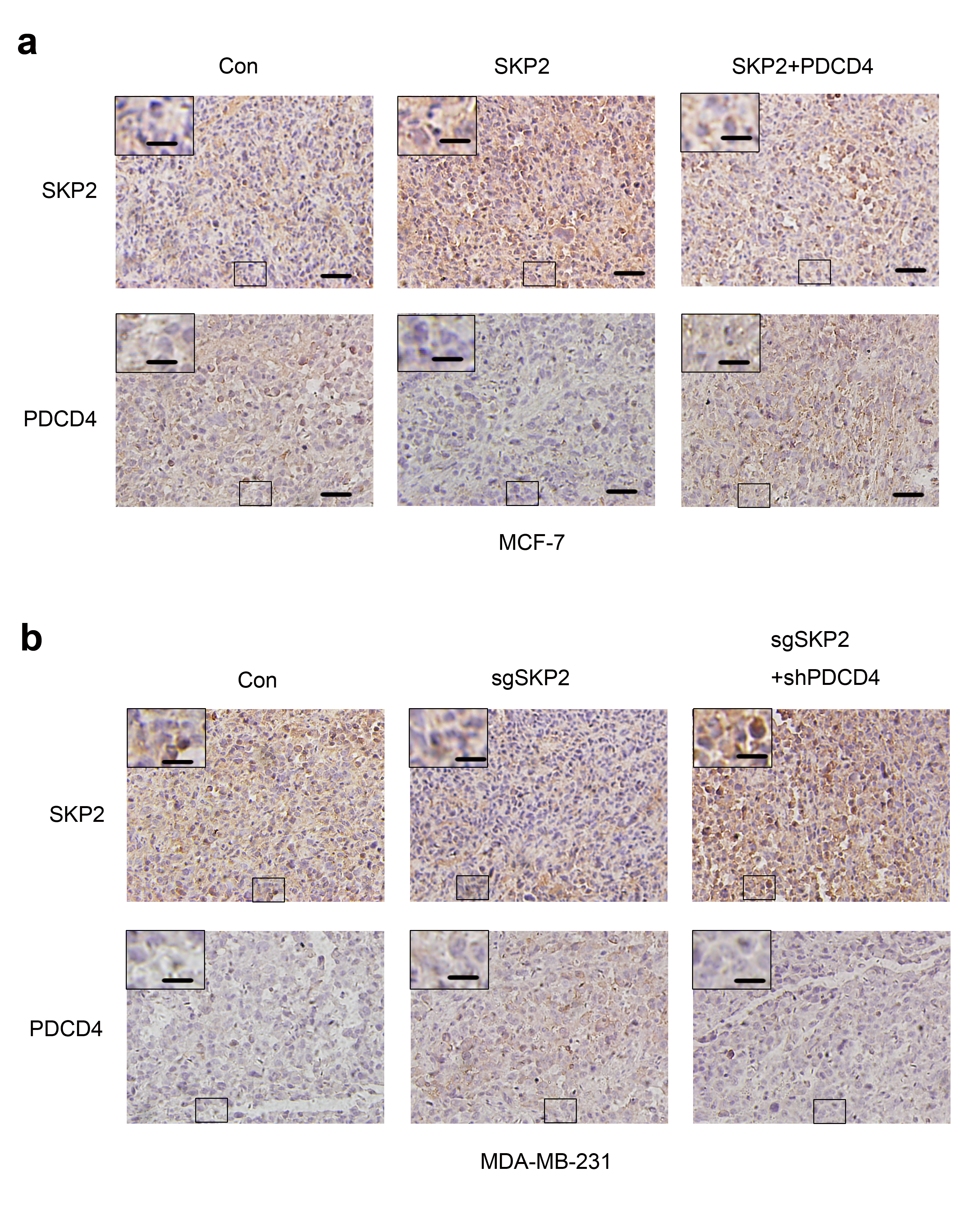

Supplement: Supplementary file 3 — Figure S3. SKP2 and PDCD4 expression in breast tumors from nude mice were detected by immunohistochemical staining. (a) Breast tumors from MCF-7-Con, MCF-7-SKP2 and MCF-7-SKP2 with PDCD4 stable expression cells were harvested from nude mice at 6 weeks for SKP2 and PDCD4 staining by IHC and quantitated (Scale bars, 50 um, Scale bars inside the box, 20 um). Data represent the mean ± SEM of three independent experiments. Student’s t-test used: *P < 0.05; **P < 0.01. (b) Breast tumors from MDA-MB-231-Con, MDA-MB-231-sgSKP2 and MDA-MB-231-sgSKP2 with shPDCD4 stable expression cells were harvested from nude mice at 6 week for SKP2 and PDCD4 staining by IHC and quantitated (Scale bars, 50 um, Scale bars inside the box, 20 um). Data represent the mean ± SEM of three independent experiments. Student’s t-test used: *P < 0.05; **P < 0.01. (TIF 31660 kb) [file 13046_2019_1069_MOESM3_ESM.tif]

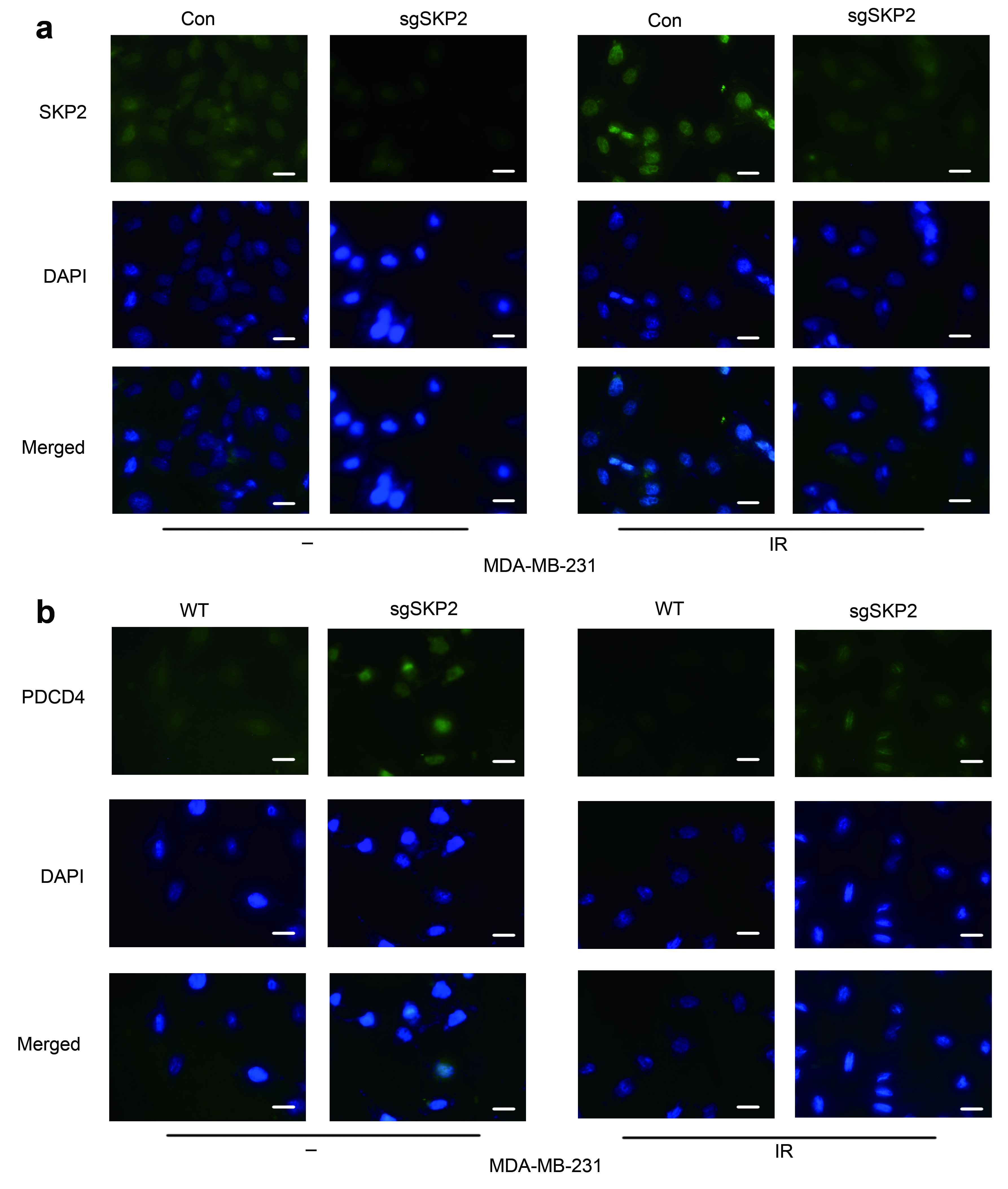

Supplement: Supplementary file 4 — Figure S4. PDCD4 is upregulated in MDA-MB-231-sgSKP2 cells after radiation. (a) SKP2 expression of MDA-MB-231-Con and MDA-MB-231-sgSKP2 cells before and after radiation is determined by immunostaining (Scale bars, 25 um). (b) PDCD4 expression of MDA-MB-231-Con and MDA-MB-231-sgSKP2 cells before and after radiation is determined by immunostaining (Scale bars, 25 um). (TIF 39287 kb) [file 13046_2019_1069_MOESM4_ESM.tif]

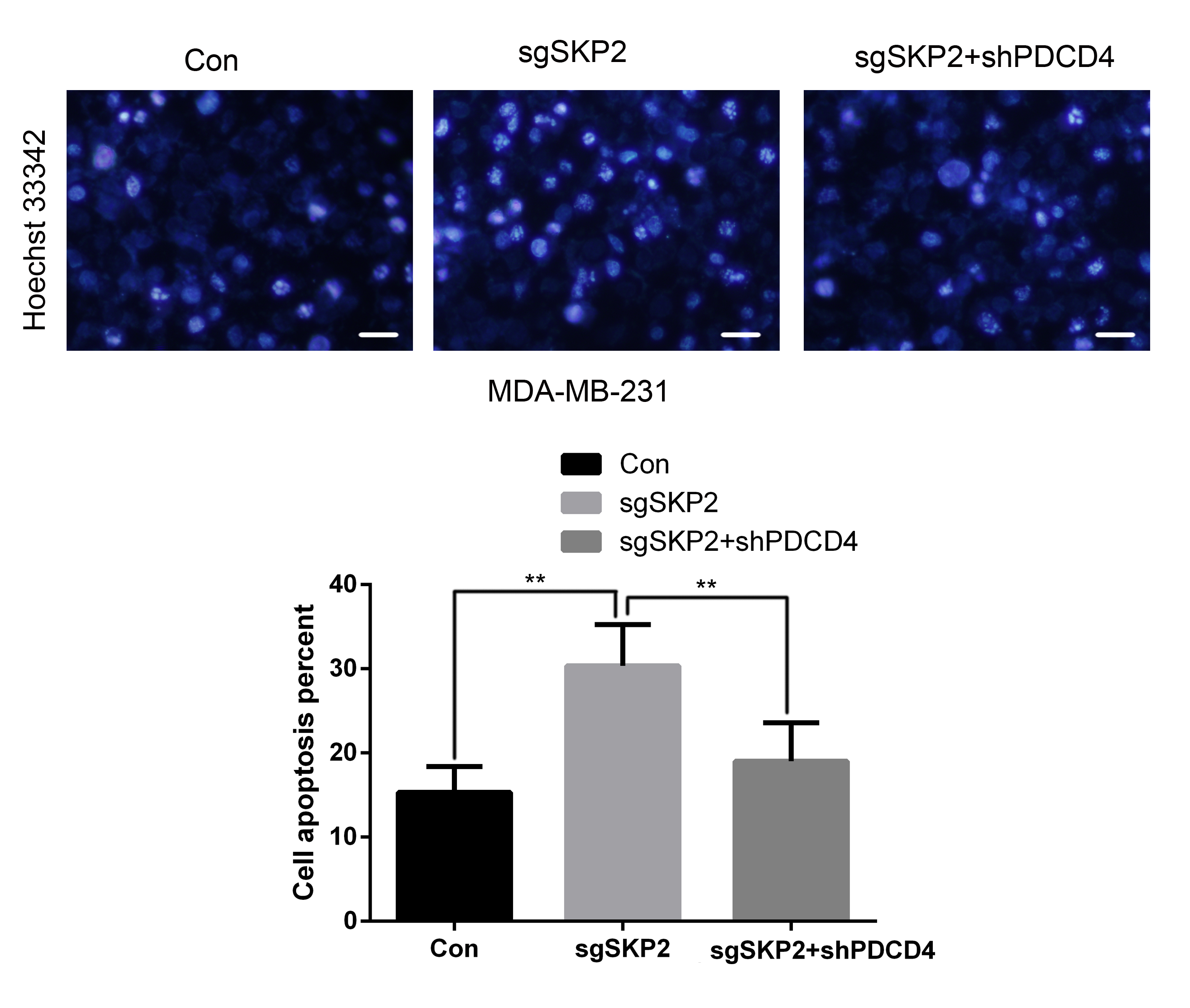

Supplement: Supplementary file 5 — Figure S5. SKP2 promotes cell apoptosis by inhibiting PDCD4 in MDA-MB-231 cells. Induction of apoptosis was determined by by Hoechst 33342 staining after 24 h cells were treated radiation (6GY) in MDA-MB-231-Con, MDA-MB-231-sgSKP2 and MDA-MB-231-sgSKP2 with shPDCD4 stable expression cells (Scale bars, 50 um). Data represent the mean ± SEM of three independent experiments. Student’s t-test used: *P < 0.05; **P < 0.01. (TIF 21910 kb) [file 13046_2019_1069_MOESM5_ESM.tif]

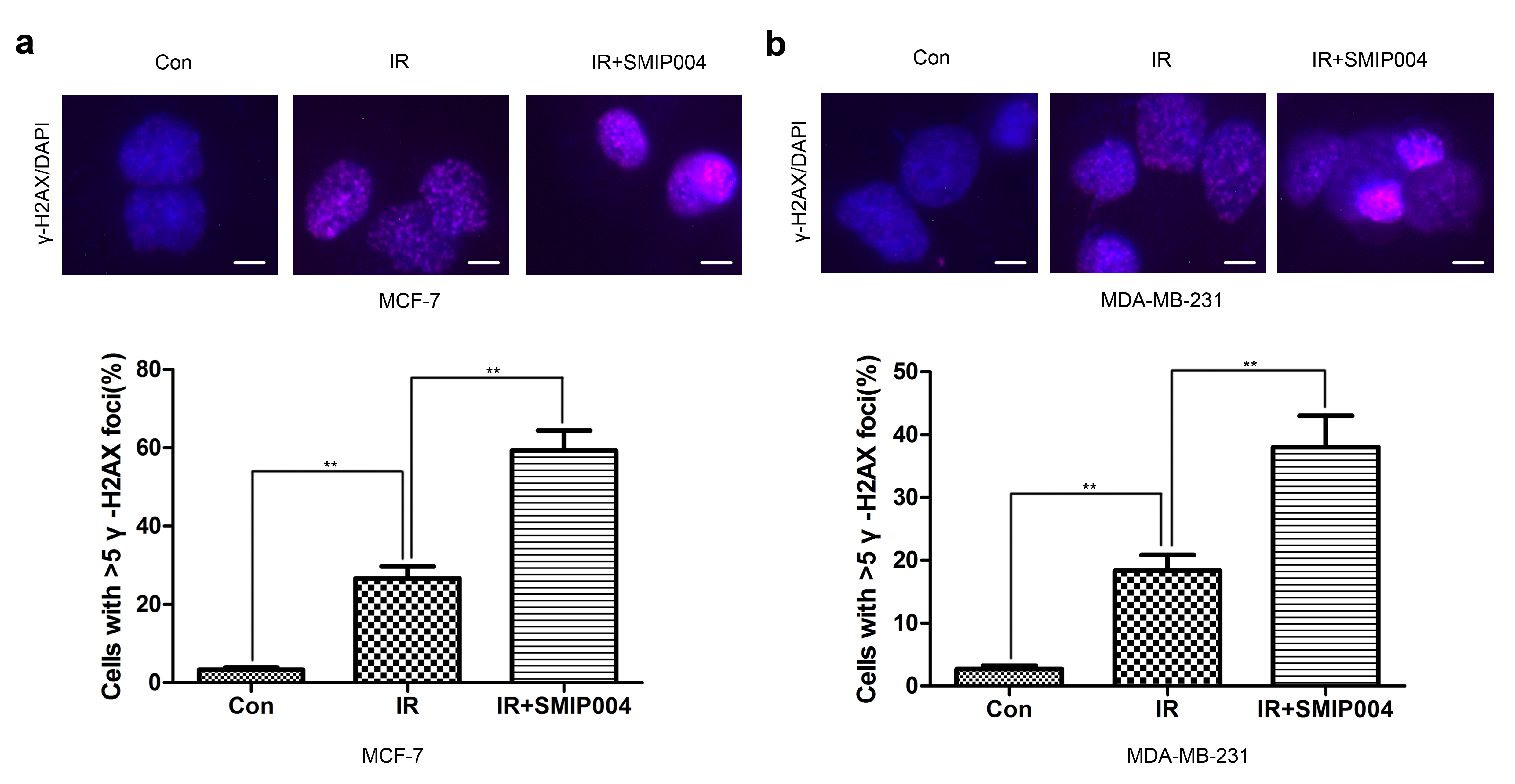

Supplement: Supplementary file 6 — Figure S6. SMIP004 promotes breast cancer cell apoptosis after radiation. (a, b) MCF-7 or MDA-MB-231 cells were untreated or treated with SMIP004 (40 μM) for 24 h, then untreated or treated with radiation (6GY). After 1 h, γ-H2AX expression is determined by immunostaining. DSBs were determined by analysis of γ-H2AX by immunostaining (Scale bars, 5 um). Data represent the mean ± SEM of three independent experiments. Student’s t-test used: *P < 0.05; **P < 0.01. (TIF 10302 kb) [file 13046_2019_1069_MOESM6_ESM.tif]

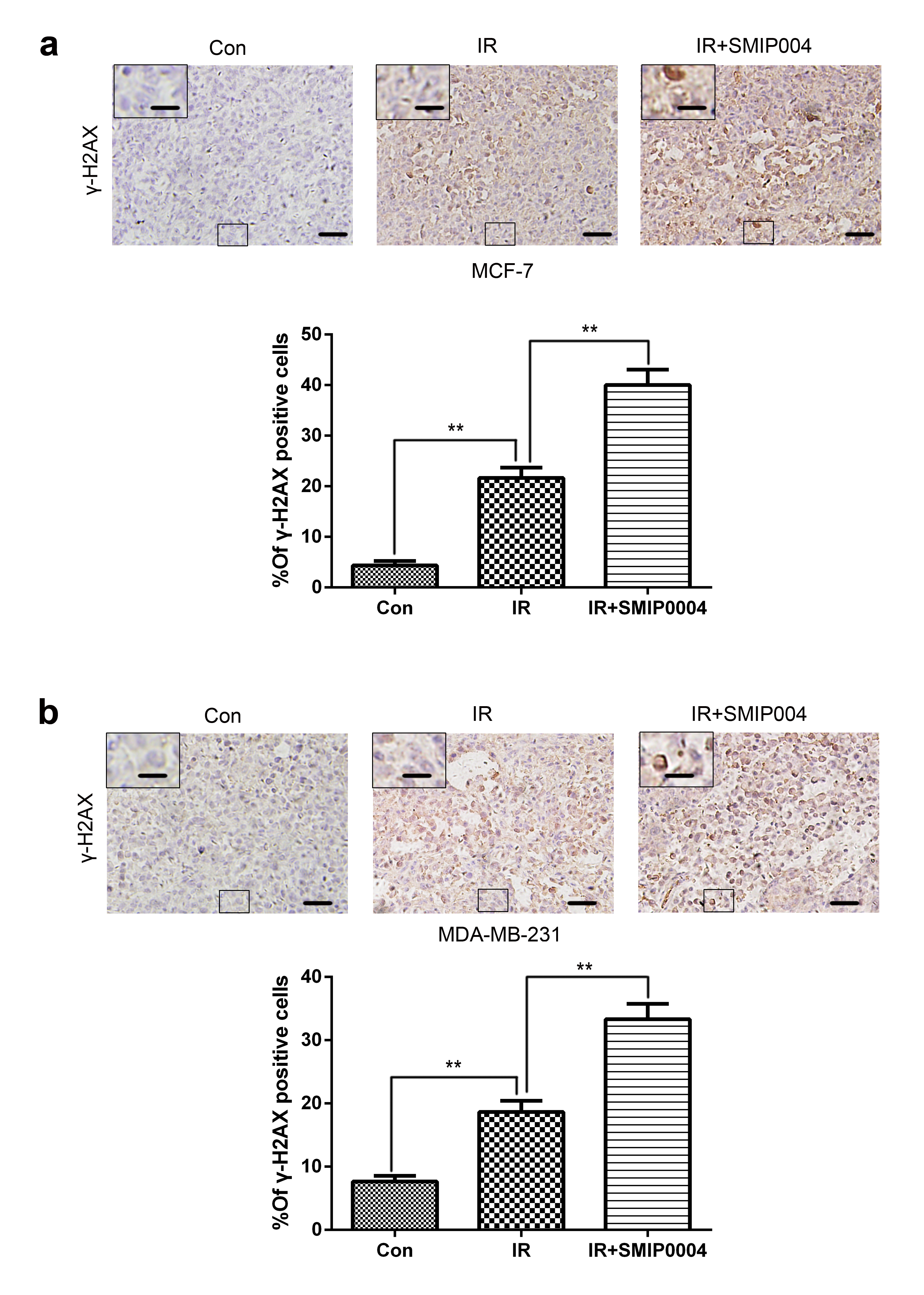

Supplement: Supplementary file 7 — Figure S7. γ-H2AX expression in breast tumors from nude mice were detected by immunohistochemical staining. (a) Breast tumors from MCF-7 cells treated or untreated with SMIP004 or radiation were harvested from nude mice at 6 week for γ-H2AX staining by IHC and quantitated (Scale bars, 50 um, Scale bars inside the box, 20 um). Data represent the mean ± SEM of three independent experiments. Student’s t-test used: *P < 0.05; **P < 0.01. (b) Breast tumors from MDA-MB-231 cells treated or untreated with SMIP004 or radiation were harvested from nude mice at 6 week for γ-H2AX staining by IHC and quantitated (Scale bars, 50 um, Scale bars inside the box, 20 um). Data represent the mean ± SEM of three independent experiments. Student’s t-test used: *P < 0.05; **P < 0.01. (TIF 17751 kb) [file 13046_2019_1069_MOESM7_ESM.tif]
